# Modified Flavonoids with Diamines and Polyamines Provide Enhanced Fluorescence and Antimicrobial Activity

**DOI:** 10.3390/ijms27010253

**Published:** 2025-12-25

**Authors:** Sevasti Matsia, Athanasios Salifoglou

**Affiliations:** Laboratory of Inorganic Chemistry and Advanced Materials, School of Chemical Engineering, Aristotle University of Thessaloniki, 54124 Thessaloniki, Greece

**Keywords:** flavonoid derivatives, Schiff base, naringin, naringenin, diamines, polyamines, fluorescence, antibacterial activity

## Abstract

Development of new biologically active materials based on natural products has, over the years, attracted considerable attention due to their effectiveness in human health and disease. Polyphenolic compounds, particularly flavonoids, provide a wide range of health benefits, including antioxidant, anti-inflammatory, anticancer, and antibacterial properties. A series of novel Schiff base derivatives of flavonoids with amino-containing linkers was successfully designed and synthesized through condensation reactions. Naringin and naringenin derivatives with diamines, including ethylenediamine (EDA), 1,3-diamino-2-propanol (DA-2-PrOH), tetramethylenediamine (TMEDA), pentamethylenediamine (PMEDA), as well as polyamines spermidine (SPD) and spermine (SPM), were synthesized and well-characterized through FT-IR, UV–Visible, ESI–MS, ^1^H and ^13^C NMR spectroscopy, and elemental analysis. The so confirmed and well-characterized derivatives were subjected to photoluminescence studies, exhibiting enhanced activity, especially for naringin-based derivatives, and quenching in some others, thus verifying the significance of chemically modifying the conjugated systems of these molecules. Their biological activity was examined in the case of their antimicrobial efficacy against two Gram (+) (*Staphylococcus aureus* and *Bacillus cereus*) and two Gram (−) (*Escherichia coli* and *Xanthomonas campestris*) bacterial strains. Antibacterial screening projected selectivity of modified flavonoids against *E. coli*, proposing new “dense” flavonoid-(poly)amine materials as multifunctional antimicrobial agents and fluorescent probes.

## 1. Introduction

Flavonoids are polyphenolic compounds, widely distributed in the plant kingdom, including fruits, vegetables, and medicinal plants [1,2,3,4,5]. They enter a broad spectrum of applications, including use in natural dyes [6,7], cosmetics and skin care products [8,9], and anti-wrinkle skin agents [10]. The most remarkable applications of these polyphenolic compounds, however, are encountered in the field of medicine. All subclasses of flavonoids are well-known for their pharmacological importance, especially for their antioxidant, anti-inflammatory, anticancer, and antibacterial properties [11,12,13]. A considerable number of flavonoids, among most known phytochemicals, has been reported for their strong antibiotic effects [14,15,16,17]. In this respect, antibacterial activity is, in most cases, attributed to their ability to disrupt bacterial membranes, chelate essential metal ions, and further interfere with nucleic acid synthesis [18,19,20]. This specific ability to interact with biomolecules and metal cofactors renders them among the most promising candidates for the development of novel antimicrobial agents.

**Scheme 1:**
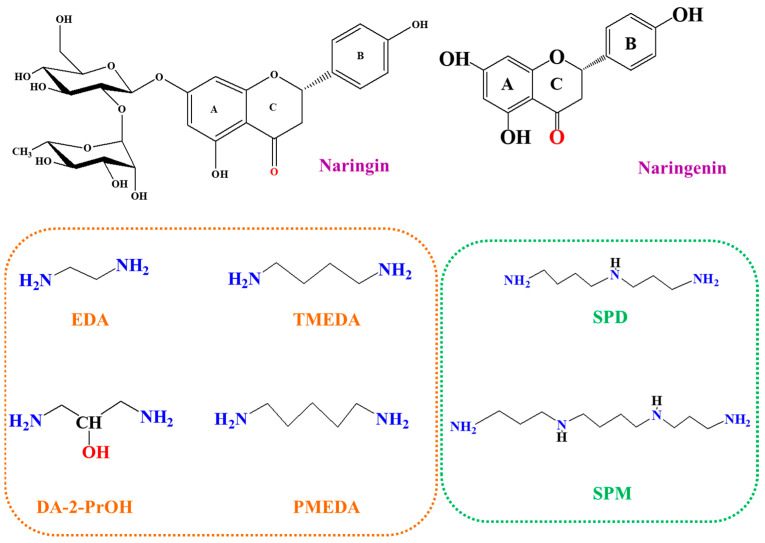
Flavonoids and N-terminal linkers implemented in this study.

In recent years, naringin and naringenin have attracted considerable interest in biomedical applications due to their broad spectrum of physicochemical and biological potential, arising from their structural characteristics and flexibility toward chemical modification [21,22]. Naringin or flavanone-7-O-glycoside is the glycoside form of the flavonoid naringenin or 4′,5,7-trihydroxyflavan-4-one. As shown in Scheme 1, naringin consists of naringenin aglycone, bearing two sugar units (neohesperidose) appended to ring A via a glycosidic linkage. Implicitly, the structure of naringenin is simpler, lacking the sugar moiety that naringin possesses. They both belong to the subclass of flavanones, bearing 15 carbon atoms in a three aromatic ring core structure (A, B, C), and apart from the fact that they exhibit structural differences, they also possess distinct physicochemical and biological profiles. In fact, there have been previous reports [23,24,25] emphasizing their ability to inhibit the growth of Gram (−) and Gram (+) bacteria, with their properties further compared to known antibiotics. Noteworthy in that respect is the antibiotic activity of flavonoids, following a different mechanism of action compared to common antibiotics, involving cell wall disruption, protein, and nucleic acid synthesis as well as biofilm control. Furthermore, due to the presence of the aromatic backbone and the carbonyl group in their structure, they exhibit π–π* and n–π* electronic transitions, thus being competitive candidates in optical applications as well [26,27].

Despite these research advances, involving independent photophysical studies or therapeutic potential, the combination of photoluminescence and antibacterial functionalities in a single flavonoid-based system is particularly attractive due to the concurrent exemplification of distinct chemical and biological properties. To that end, the idea emerged in this study to combine both photoluminescence and antibacterial properties, thus promoting multifunctional materials in the field of theranostics, fluorescent probes, bioimaging with antibacterial action, or even food-packaging antimicrobial sensors. This dual focus on natural product-containing hybrid molecules (a) incorporates the design and synthesis of new Schiff base derivatives of flavonoids, thereby targeting their biochemical profile (rather than creating metal complexes [28,29,30,31] or single-site modifications reported so far [32]), (b) seeks delineation of their physicochemical profile, extending to fluorescence properties that correlate with biological activity, instead of focusing only on specific properties, and (c) enables a systematic comparison between glycosylated (naringin) and aglycone (naringenin) moieties of flavonoids in the emerging (poly)amine derivatives under identical conditions to unravel the role of the sugar moiety in a combined photophysical-biological profile. To address this specific task, molecules that facilitate Schiff base condensation reactions with flavonoids have been selected, including diamines and polyamines. Such approaches (involving Schiff base condensation) are investigated and reported [33] to give rise to new molecules, bearing characteristic imine groups (–RC=N–), which in most cases show enhanced fluorescence properties due to the introduction of a new π-system conjugated to aromatic rings [34,35]. Furthermore, the emerging C=N double bond is expected to extend the π-conjugated system of the flavonoid moiety, thus facilitating *π*–π* and intramolecular charge transfer transitions (due to the ability to act as donor/acceptor bridges) [36], which in turn may provide enhanced emission intensity and bathochromic shifts. In many Schiff bases of aromatic nature, the imine nitrogen also participates in hydrogen bonding and metal ion coordination, with DNA and proteins further stabilizing excited states and modulating emission properties [37,38,39].

Key role in the described effort play the linkers selected (diamines/polyamines), which, as shown in Scheme 1, are expected to modulate and configure the physicochemical and biological properties of the arising hybrid products [40,41,42]. Diamines and polyamines are such biologically relevant molecules found in living cells, promoting stabilization of DNA, participation in cell growth, and stress responses. At physiological pH values, their cationic nature facilitates electrostatic interactions with negatively charged biomolecules, such as nucleic acids and bacterial membranes, thus attesting to their biological relevance and activity profile(s).

Variation in the chain length and number of amine moieties in the (poly)amine linkers project their ability to modulate lipophilicity, solubility, hydrogen bonding, and metal chelation. In that sense, the selection of diamines and polyamines as linkers in the proposed chemical reactivity toward polyphenols is particularly strategic. Short diamines, like EDA, are expected to provide compact structures with strong π–π coupling, whereas bulkier ones (TMEDA, SPM) will introduce different electron-donating capabilities through their nitrogen atoms. Consequently, parameterization of the proposed chemical reactivity of selected polyphenol molecules with variably yet distinctly structured (poly)amines signifies the logic of the undertaken chemical reactivity and targets newly derived ternary organic derivatives that (a) exemplify the emergence of new “denser” molecular species of natural origin that could concentrate antibacterial and antibiotic activity in a single hybrid molecule, (b) possess distinctly differentiated physicochemical profiles from their predecessor starting molecules, and (c) exhibit both optically unique and biologically competent antibacterial activities, collectively setting the stage for custom-made multifunctional materials with applications in abiotic and biological systems.

## 2. Results

### 2.1. Synthesis of Modified Flavonoids Through Schiff Base Reactivity

**Scheme 2:**
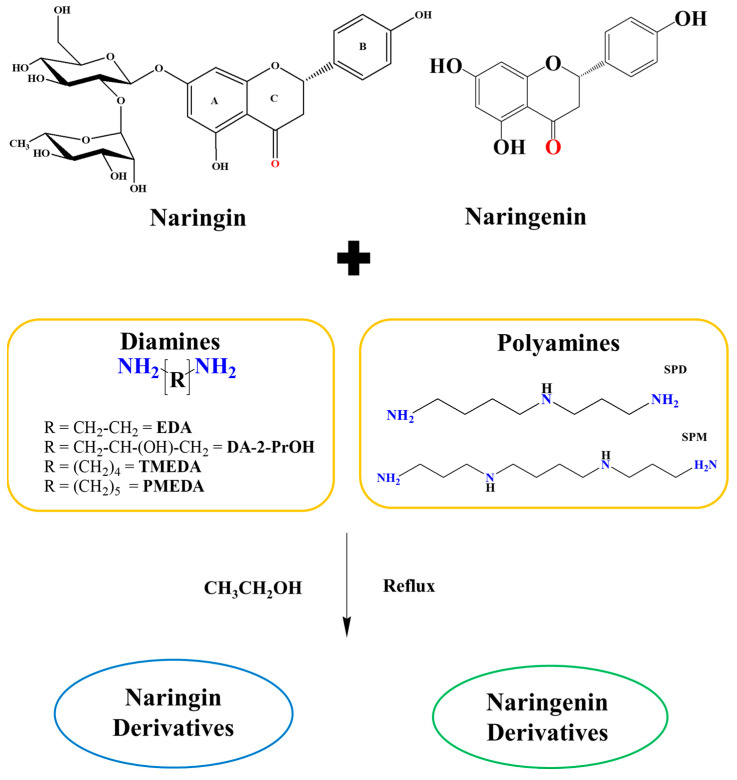
General reaction synthesis for (poly)diamine naringin and naringenin derivatives.

**Scheme 3:**
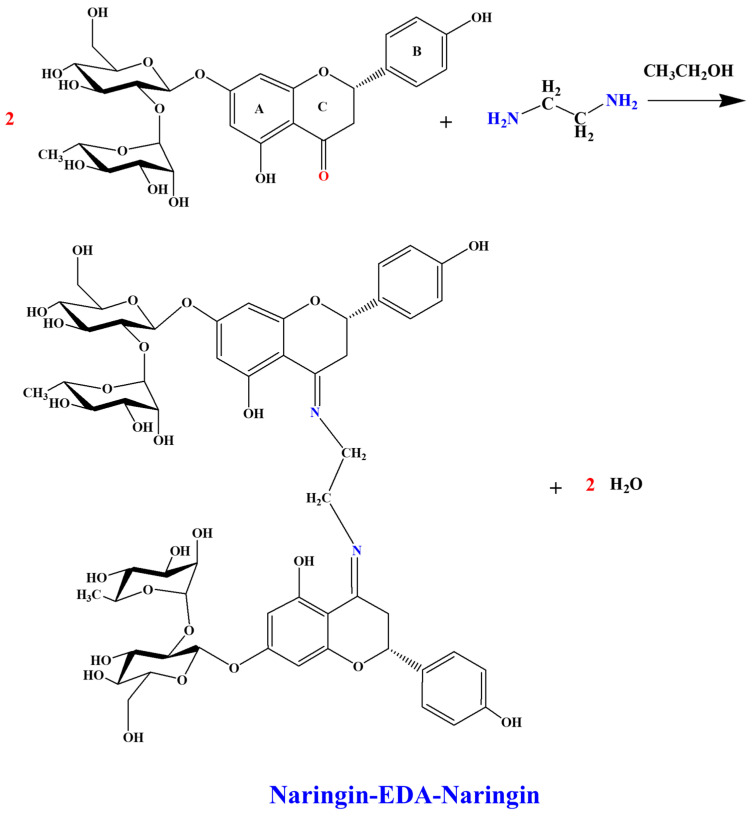
Naringin Schiff base reactivity with EDA.

Modified flavonoids (flavonoid derivatives) have been synthesized through Schiff base condensation reactions between naringin or naringenin with diamines/polyamines in alcoholic media, with a molar ratio 2:1, by heating the appropriate mixtures overnight. A 2:1 flavonoid/diamine molar ratio was chosen after preliminary screening, involving variable molecular stoichiometries (2.5:1 and 3:1 molar ratios). The selected optimized ratio minimizes singly derivatized flavonoid byproducts, while enabling straightforward isolation of the di-flavonoid hybrid moieties. The general chemical reaction leading to the isolation of flavonoid derivatives is shown in Scheme 2. Specifically, the reaction of naringin with EDA is shown in Scheme 3, whereas all other derivatives are depicted in Reactivity Schemes S1–S11.

### 2.2. FT-IR Spectroscopy

FT-IR spectra of all modified flavonoids have been compared to those of free naringin and naringenin patterns. Specifically, for naringin-based Schiff base derivatives, a shifted characteristic broad resonance emerges (Table 1) at 3417 cm^−1^ (for free naringin) related to O-H stretching vibrations. In free naringin, C-H stretching vibrations of saturated carbons appear at 2926 cm^−1^, shifted in the spectra of the modified flavonoids, as shown in Table 1. The most important resonance related to carbonyl stretching vibrations is shown at 1649 cm^−1^, shifted to lower wavenumbers and attributed to C=N vibrations of the modified flavonoids. In the range 1205–1043 cm^−1^, resonances are attributed to the C-O stretching vibrations of aromatic ether and fatty ether bonds. The corresponding range of absorptions appears shifted in the spectra of the modified materials. Resonance peaks at 888 and 819 cm^−1^, in all cases being present, attest to the existence of a glycosidic bond and C-H out-of-plane bending vibrations of the B-ring. In the case of naringenin derivatives, absorption peaks are shown in Table 1. Characteristic O-H stretching vibrations, shown at 3285 and 3118 cm^−1^ for naringenin, were observed as shifted and broader resonances for the modified flavonoids. Resonances at 2911 cm^−1^ for C-H vibrations are also shifted in the spectra of all materials. Furthermore, C=O absorptions at 1633 cm^−1^, in the FT-IR pattern of naringenin, disappeared, and a new resonance related to C=N stretching vibrations emerged at lower energies, thus indicating the presence of a new moiety (C=N) due to the chemical modification imposed by the reacting diamines and polyamines [43,44,45,46]. The aforementioned spectral shifts confirm the successful condensation and Schiff base formation.

### 2.3. UV-Visible Spectroscopy

**Figure 1:**
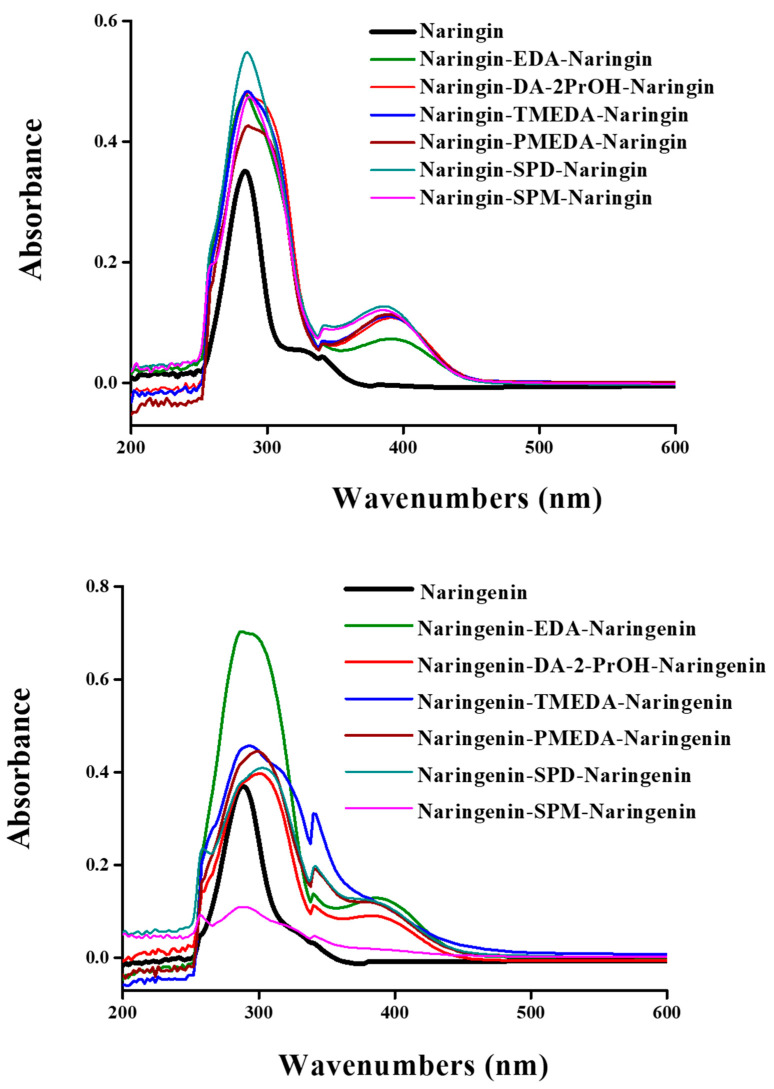
Comparative UV-Visible spectra of neat and modified flavonoids at 2 × 10^−5^ M in DMSO solution.

UV-Visible spectra of all generated materials were recorded in DMSO, in the concentration range from 10^−3^ to 10^−5^ M, via serial dilutions. Comparative spectra of all modified flavonoids with naringin and naringenin are shown in Figure 1. Naringin-based UV-Visible spectra exhibit one absorption band at 284 nm for neat naringin, a feature also present in modified materials, shown with higher intensity correlated to the benzoyl system. The other absorption, present as a shoulder at 320 nm for the neat flavonoid, appears at ~390 nm. It possesses higher intensity for modified derivatives and reflects π→π* electronic transitions associated with the extended conjugation of the flavonoid backbone (B-ring). Moving on to naringenin derivative spectra, they also show the same pattern. Specifically, a high intensity band at 288 nm and a shoulder at 324 nm are attributed to π→π* transitions of the benzoyl (A-ring) and cinnamoyl system (B-ring), respectively. The so-modified flavonoids of naringenin showed a higher intensity band (except for the ternary Naringenin-SPM-Naringenin) at 287–302 nm and a broad band at 380–390 nm, displaying a bathochromic shift [47,48,49]. Molecular absorption coefficient values are shown in Table 1 for neat flavonoids and derivatives thereof. Spectral variations, including enhancement and shifts in absorption features compared to free flavonoids, confirm the successful modification of flavonoids, thereby hinting at electronic interactions between flavonoid chromophores and the polyamine moieties.

### 2.4. ESI-MS Profile and Features

The ESI-MS spectra of naringin and naringenin derivatives were recorded at a concentration of 10^−4^ M, in methanolic solutions, with the appropriate patterns shown in Figures S1 and S2. The following molecular ions are depicted and shown, based on codes of flavonoid derivatives (M1–M12), as explained in Section 4.2.1: M1_1 = [M1]^+^ = [C_56_H_68_N_2_O_26_]^+^, *m/z* = 1185.4489 (z = 1); M1_2 = [M1-C_27_H_33_O_13_]^+^ = [C_29_H_35_N_2_O_13_+4H]^+^ = [C_29_H_39_N_2_O_13_]^+^, *m/z* = 623.2611 (z = 1); M2_1 = [M2]^+^ = [C_57_H_70_N_2_O_27_]^+^, *m/z* = 1215.4283 (z = 1); M3_1 = [M3]^+^ = [C_58_H_72_N_2_O_26_]^+^, *m/z* = 1213.4441 (z = 1); M4_1 = [M4]^+^ = [C_59_H_74_N_2_O_26_]^+^, *m/z* = 1227.4692 (z = 1); M5_1 = [M5]^+^ = [C_61_H_79_N_3_O_26_]^+^, *m/z* = 1270.5035 (z = 1); M6_1 = [M6]^+^ = [C_64_H_86_N_4_O_26_]^+^, *m/z* = 1327.5599 (z = 1); M7_1 = [M7+H]^+^ = [C_32_H_28_N_2_O_8_+H]^+^, *m/z* = 569.1941 (z = 1); M8_1 = [M8+H]^+^ = [C_33_H_30_N_2_O_9_+H]^+^, *m/z* = 599.1998 (z = 1); M9_1 = [M9+H]^+^ = [C_34_H_32_N_2_O_8_+H]^+^, *m/z* = 597.2238 (z = 1); M10_1 = [M10+H]^+^ = [C_35_H_34_N_2_O_8_+H]^+^, *m/z* = 611.2289 (z = 1); M11_1 = [M11+H]^+^ = [C_37_H_39_N_3_O_8_+H]^+^, *m/z* = 654.2775 (z = 1); M12_1 = [M12]^+^ = [C_40_H_46_N_4_O_8_]^+^, *m/z* = 710.3031 (z = 1). All of the aforementioned species are present in ESI-MS spectra (Figures S1 and S2), with *m/z* values having been used for the estimation of absolute error values compared to the simulated ones, as shown in Table S1.

### 2.5. ^1^H and ^13^C NMR Spectroscopy

**Figure 2:**
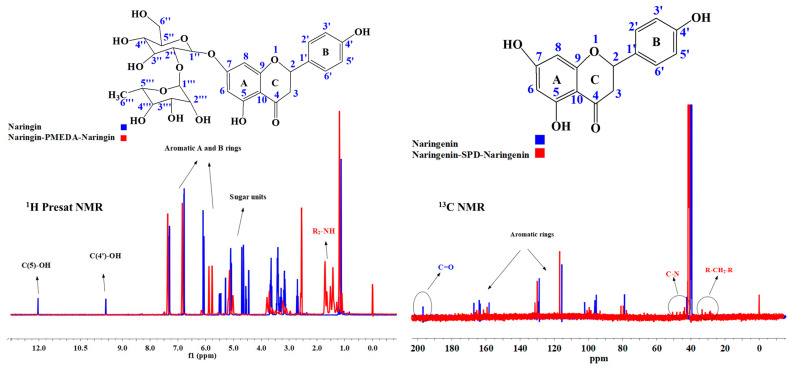
Representative ^1^H PRESAT and ^13^C-NMR patterns of modified flavonoids compared to free flavonoids collected in DMSO-d^6^.

The structures of the newly synthesized Schiff base derivatives of naringin and naringenin were further confirmed by ^1^H- and ^13^C-NMR spectroscopy. In Figure 2, representative spectra of ^1^H (PRESAT) and ^13^C NMR patterns of a species are shown, revealing shifts in specific groups compared to those of free flavonoids.

Specifically, in the case of the ^1^H-NMR spectra of modified Naringin-PMEDA-Naringin, there are differences compared to free naringin, as follows: two distinct signals at 12.04 ppm and 9.59 ppm, related to the phenolic groups of A (C(5)-OH) and B rings (C(4′)-OH) of free naringin, dissapeared or broadened (especially the one at ~9.50 ppm) in all modified flavonoids. This observation is linked to their involvement in hydrogen bonding during Schiff base formation or even condensation with the selected amine linkers [50].

In the region 6.00–7.50 ppm, corresponding to the aromatic A and B ring hydrogens, all signals corresponding to the B ring are not shifted, whereas the signal at 6.10 ppm (C(6)-H and C(8)-H) is shifted upfield in all derivative spectra, thus verifying that the undertaken derivatization affects the aromatic A ring of the flavonoids. Further, it was concluded that the signals observed in the range 5.10–5.90 ppm and 3.00–4.00 ppm, corresponding to the anomeric protons of glucose and ramnose units, are shown to be shifted downfield due to stereoelectronic changes, following introduction of the C=N group. The hydrogen signals, corresponding to C(2)-H and C(3)-H, are depicted as a multiplet peak at 2.50 ppm. In the range 1.00–1.80 ppm, the peaks appear also shifted and they are observed through the presence of methyl groups present in the sugar units. The same spectral region provides extra peaks linked to the existence of more -CH_2_- groups being present from the diamines and polyamines. As for the naringenin derivatives (Figures S3–S13), in the ^1^H-NMR spectra, there is dissapearance/emergence of broad signals for the three peaks linked to the OH groups of the aromatic rings shown at 12.13 ppm for C(5)-OH, 10.80 ppm for C(7)-OH, and at 9.61 ppm for C(4′)-OH. Singlet resonances in the range 6.00–8.00 ppm appeared to be unaffected, except for the signal at 6.00 ppm, which is related to resonances of the A ring (shown shifted downfield). New peaks close to 1.00–2.00 ppm verify the incorporation of the diamine/polyamine linker in the derivative flavonoids [51].

Moving on to the ^13^C-NMR spectra, it is shown that the singlet peak for C=O, shown at 197.49 ppm for free naringin and at 196.92 ppm for naringenin, has disappeared in all flavonoid derivatives. As a result, upfield of the peaks in the range 115.00–165.00 ppm (aromatic rings), at ~170.00 ppm, there is a singlet resonance, which is characteristic of C=N bonds. The presence of diamines/polyamines in the modified flavonoids was also verified due to the existence of singlet peaks in the range 20.00–45.00 ppm, characteristic for aliphatic CH_2_ carbons, as shown in Figure 2 and Figures S3–S13 [52].

### 2.6. Fluorescence Activity

**Figure 3:**
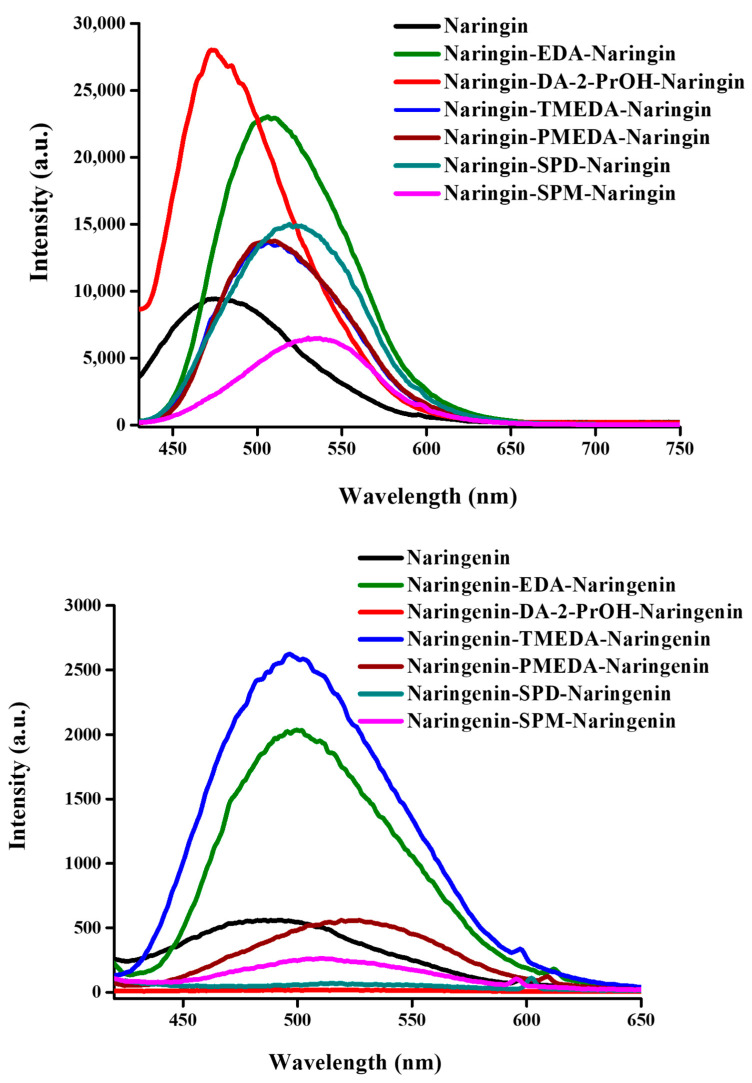
Comparative fluorescence spectra of naringin and naringenin derivatives with neat flavonoids in the solid state.

Solid-state fluorescence spectra of naringin, naringenin, and their derivatives were recorded at room temperature. The emerging patterns are provided in Figure 3, through comparative graphs for naringin and naringenin-based molecules, adjusted to project a common molar basis of comparison. For naringin derivatives, an enhancement of the intensity for all derivatives (except Naringin-SPM-Naringin) is observed compared to the free flavonoid. Moreover, an emission shift is observed in most of the Schiff base derivatives. Specifically, free naringin exhibits an emission band at 477 nm (λ_ex_ 396 nm) [53]. Derivatives of naringin with diamines and polyamines show an emission band at 506 nm (λ_ex_ 397 nm) for Naringin-EDA-Naringin, 508 nm (λ_ex_ 397 nm) for Naringin-DA-2-PrOH-Naringin, 508 nm (λ_ex_ 410 nm) for Naringin-TMEDA-Naringin, 506 nm (λ_ex_ 397 nm) for Naringin-PMEDA-Naringin, 522 nm (λ_ex_ 396 nm) for Naringin-SPD-Naringin, and 535 nm (λ_ex_ 397 nm) for Naringin-SPM-Naringin. As far as naringenin derivatives, a quite different pattern is revealed. It can be seen that EDA and TMEDA derivatives showed enhanced intensity compared to free naringenin, while all of the other derivatives provide the same or quenching effect. Furthermore, a slight shift was unraveled in most of the derivatives. Concretely, free naringenin shows an emission band at 512 nm (λ_ex_ 400 nm) [54]. Schiff base derivatives exhibit an emission band at 500 nm (λ_ex_ 407 nm) for Naringenin-EDA-Naringenin, 503 nm (λ_ex_ 397 nm) for Naringenin-DA-2-PrOH-Naringenin, 500 nm (λ_ex_ 397 nm) for Naringenin-TMEDA-Naringenin, 523 nm (λ_ex_ 405 nm) for Naringenin-PMEDA-Naringenin, 521 nm (λ_ex_ 400 nm) for Naringenin-SPD-Naringenin, and 513 nm (λ_ex_ 396 nm) for Naringenin-SPM-Naringenin. Overall, the observed red-shift (bathochromic shift) of the emission maxima in most Schiff base derivatives, compared to free flavonoids, is consistent with the conjugation of the amine terminals to the flavonoid backbone (vide infra). These findings demonstrate that Schiff base modification constitutes an effective strategy to change, modulate, and in some cases, enhance the photoluminescence properties of flavonoids.

### 2.7. Antibacterial Properties

The well-defined and well-characterized Schiff base derivatives have been further investigated for their antibacterial potential in Gram (−) (*E. coli* and *X. campestris*) and Gram (+) (*S. aureus* and *B. cereus*) bacterial strains. The disk diffusion method was employed, and all derivatives were investigated in a concentration-dependent manner along with their appropriate starting materials (free flavonoids and diamine/polyamine) as controls. The method applied has been chosen due to low solubility of the materials investigated in aqueous media and in LB Broth media following dissolution in DMSO. The final results were adjusted on a molar basis in order to have meaningful comparisons. Overall, the minimum inhibitory concentration (MIC), which is responsible for portraying antibacterial effects, is expressed as mg/cm^2^, and in every case, the Zone Of Inhibition (ZOI) value was determined. The antibacterial effect results in the case of the Gram (−) bacterium of *E. coli* for naringin derivatives as well as the controls are shown in Table 2.

In the broad spectrum of biological investigation tests run, it is shown that Schiff base derivatives of naringin provide high antibacterial activity, whereas controls (naringin and diamines/polyamines) show no effect against *E. coli*. Specifically, derivatives with EDA, DA-2-PrOH, and polyamines show almost the same ZOI (~26.0 ± 0.2 mm), with the amount needed being approximately the same (2.0–3.0 mg/(7.1–11 mg/cm^2^)) except for the derivative with EDA (5.0 mg/(18 mg/cm^2^)). Τhe other two derivatives with diamines TMEDA and PMEDA, for the same amount (2.0 mg/7.1 mg/cm^2^), exhibit a stronger antibacterial effect against *E. coli*, bearing a ZOI at 38.3 ± 0.3 mm and 29.8 ± 0.2 mm, respectively. Moving on to naringenin derivatives (Table 3), it is shown that the amount needed to achieve antibacterial activity against *E. coli* is higher than that for naringin-based derivatives, thus highlighting a different profile.

**Figure 4:**
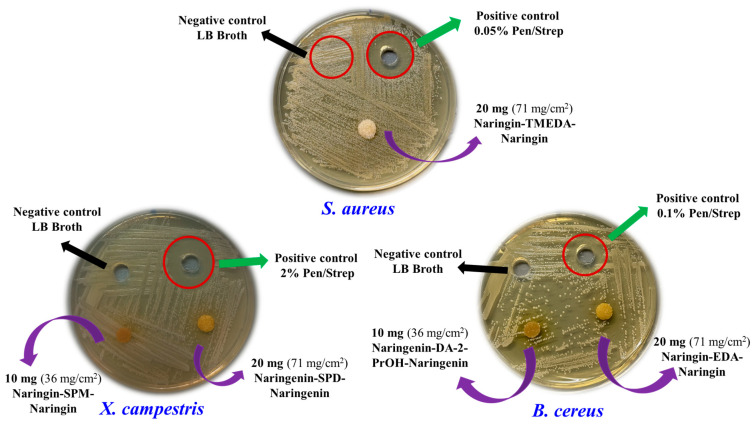
Bacterial strains showing no zone of inhibition in the presence of Schiff base-modified flavonoids.

For all derivative molecules, an amount of 10 mg (36 mg/cm^2^) is needed to reach the ZOI at 23.3 ± 0.2 mm for Naringenin-EDA-Naringenin, 17.3 ± 0.1 mm for Naringenin-DA-2-PrOH-Naringenin, 19.9 ± 0.1 mm for Naringenin-TMEDA-Naringenin, 16.5 ± 0.3 mm for Naringenin-PMEDA-Naringenin, 26.9 ± 0.1 mm for Naringenin-SPD-Naringenin, and 21.5 ± 0.1 mm for Naringenin-SPM-Naringenin. Derivatives with EDA and polyamines exhibited the highest ZOI, while comparison to free components reveals that naringenin shows no inhibition activity. The free diamines/polyamines exhibit antibacterial activity, starting from ~1.0 mg (3.6 mg/cm^2^) up to ~9.0 mg (32 mg/cm^2^). In the case of naringin derivatives, it is confirmed that antibacterial activity arises from the Schiff base framework formation rather than the amines themselves, as in the naringenin-based molecules [55,56,57]. All of the aforementioned Schiff base derivatives show no inhibition on the other tested bacteria (Figure 4), including *S. aureus*, *B. cereus*, and *X. campestris*, being selective only in the case of the *E. coli* strain.

## 3. Discussion

### 3.1. Synthetic Flavonoid Derivatives and Physicochemical Profile

Polyphenolic compounds, and especially flavonoids, are among the most widely studied phytochemicals due to their flexible structural skeleton and the broad spectrum of exerted biological activities, including antioxidant, anticancer, anti-inflammatory, and antimicrobial effects. However, their physicochemical limitations (solubility, rapid metabolism, and relatively weak photoluminescence activity), in most cases, restrict their application in many fields, including the medical field. Driven by the need to generate hybrid molecular species of natural origin with enhanced bioactivity, we focused on chemical modification strategies targeting flavonoids (new strategies distinguishing them from the ones reported so far [28,29,30,31,32]), with representative target molecules being the intimately related naringin and naringenin species (vide infra). This specific work has managed to overcome the indigenous limitations of flavonoids by combining them with (poly)amines, thus leading to new hybrid entities that concentrate flavonoids in a single molecular assembly. The adopted approach gives rise to new materials that exhibit enhanced fluorescence properties and biological activity. Such a combination portends extended applications that essentially exemplify multifunctional materials through novel approaches and strategies, compared to targeted modifications in flavonoid chemistry reported so far (i.e., complexation of flavonoids with metal ions [28,29,30,31] and single-site modification [32]).

Among the various approaches for flavonoid chemical modification or derivatization, Schiff base condensation represents an attractive pathway. To that end, the idea of introducing an imine bond (–C=N–) to a composite flavonoid-(poly)diamine assembly projects extension to the already existing conjugated π-system of the flavonoids, thus giving rise to enhancement of fluorescence. It is also important to point out the fact that new chemically modified flavonoids stand as attractive sites for metal ion binding, hydrogen bonding, and transfer interactions, thus giving rise to new properties and activities at the biological level. In this context, the approach emphasizes the value of bridging two flavonoid units via diamine and polyamine linkers, thus yielding new bifunctional assemblies with enhanced fluorescence and selective antibacterial activity. Such a strategy enhances and demonstrates the fact that rational molecular design can generate multifunctional agents, suitable for biomedical, sensing, and antimicrobial applications. The model systems employed were two flavonoids intimately related to each other: naringin and naringenin, with the difference being the disaccharide moiety appended to the A ring in naringin compared to naringenin. By the same token, the selection of naturally significant (poly)amines as bridging ligands to abutting flavonoids was another rationally conceived selection in an effort to derive the intended hybrid species. In that respect, in all cases, Schiff base derivatives were synthesized and isolated in good yields. Due to structural differences between the two basic flavonoids, naringin derivatives showed higher yields, thereby increasing solubility in polar solvents and enhancing accessibility of emerging reaction intermediates, collectively contributing to more favorable conversion. On the other hand, naringenin, being the aglycone form, is less soluble in aqueous or mixed solvent systems, a property that may result in incomplete condensation and reduced isolated yields.

The new imine bond formation and successful condensation reaction in the produced materials were further confirmed by elemental analysis, FT-IR, and UV-Visible spectroscopies. Specifically, the appearance of a new FT-IR band around 1608–1613 cm^−1^ for naringin and 1589–1592 cm^−1^ for naringenin derivatives (shifted compared to C=O in free naringin) suggests conjugation with the amine group(s). The aromatic C=C stretches (~1500–1600 cm^−1^) remained unchanged, as were the glycosidic bond resonances (in the case of naringin), thus attesting to the flavonoid backbone remaining intact. To further validate these findings, UV-Visible spectroscopy was instrumental in showing that absorptions related to the cinnamoyl system (~320–392 nm for ring B) exhibited enhancement of absorbance and bathochromic shift (red shift), thus reflecting increased π–π* conjugation and intramolecular charge transfer due to C=N bond formation. It is worth pointing out that the enhanced UV–Visible absorption intensity of Schiff base derivatives compared to free flavonoids (for the same concentration) indicates that chemical modification increases molar absorptivity due to extended π-conjugation and charge transfer pathways, making the molecules optically more active. In the case of naringin derivatives, a higher bathochromic shift occurs compared to naringenin derivatives, thus showing that there is a more extended π-conjugation system, which provides stronger intermolecular charge transfer and more efficient light absorption. The Schiff base chemical modification led to an increased electronic delocalization and polarizability in the case of naringin derivatives. On the other hand, it was observed that the Naringenin-SPM-Naringenin derivative exhibits the lowest UV-Visible absorbance in all hybrid naringenin species with a lower molecular coefficient value. It is likely that steric hindrance and conformational flexibility, emanating through the SPM diamine linker in the hybrid species, may disrupt π–π conjugation and further favor non-radiative pathways, thereby leading to weaker electronic transitions as concluded from the ensuing fluorescence studies. All of the aforementioned observations are also verified through higher ε values than in free naringin. The same trend in ε values is also shown in naringenin derivatives, except for Naringenin-SPM-Naringenin, which exhibits the lowest values. These changes indicate that Schiff base formation increases the probability of the π–π transition associated with the aromatic system and the n–π* transition of the carbonyl/imine moieties. The red-shifted second absorption band (typically in the range 380–392 nm) can be assigned to the enhanced ICT (Intramolecular Charge-Transfer) transition, arising from conjugation between the flavonoid backbone and the electron-donating amine group.

To further enhance and support the structural reactivity profile of the newly derived materials, ESI–MS spectrometry and NMR spectroscopy were further employed. From the ESI-MS results, it is clear that all derivatives retain their structure when in solution, thus indicating stable forms. Molecular ion peaks confirm that condensation was successful, further indicating their structural integrity and purity. Overall, experimental *m/z* values are a good match to simulated values, thus providing the basis for further optical and antibacterial studies. Moreover, the aforementioned results are further compared and agree with NMR data, supporting Schiff base formation.

Collectively, the analytical and spectroscopic (FT-IR, UV-Visible, and NMR) as well as mass spectrometric (ESI-MS) data confirmed the successful synthesis of Schiff base derivatives of both naringin and naringenin with polyamines. The specific techniques not only verify the structural integrity but also highlight the influence of the imine group on the electronic properties of the flavonoid core of the ternary hybrid species. Consequently, the choice of diamine/polyamine linker in the Schiff reactivity influences both the chemical environment of the imine and the overall backbone behavior of the flavonoid molecule.

### 3.2. Photoluminescence Activity

The well-characterized materials have been further studied for their optical properties through photoluminescence activity in the solid state. Free naringin shows an emission at 477 nm, while naringenin displays an emission at 512 nm, with naringin exhibiting 20 times higher activity than its aglycone form. This phenomenon is likely due to strong non-radiative deactivation pathways in naringenin, emanating from intramolecular hydrogen bond formation between the C(5) phenolic group and the carbonyl moiety of the polyphenolic structure in the photoexcited state. This way, hydrogen bond formation facilitates excited state proton transfer and vibrational relaxation, which further act as competitive non-radiative decay channels that reduce emission intensity [58,59,60]. In contrast to that behavior, in naringin, due to the existence of a sugar moiety, there are steric effects leading to no hydrogen bond formation (the sugar moiety competes for hydrogen bond formation with the phenolic group) [61]. Their ternary derivatives with (poly)amines show, in most cases, a bathochromic shift along with an enhanced emission in the case of the naringin Schiff base-modified molecules, in contrast to only partial enhancement and a noticeable emission shift emerging in the case of the naringenin modified flavonoids. Specifically, for naringin-based derivatives, it can be seen that the shorter the linker length and complexity (EDA and DA-2-PrOH) the higher the emission band observed. For the EDA linker, a ~2.7-fold enhancement is observed, whereas for DA-2-PrOH the observed rise is ~2.3 times that of free naringin. Upon increasing the chain length of the linker, as in the case of TMEDA and PMEDA, the fluorescence emission enhancement decreases to 1.3 times that of free naringin. For the polyamines, a slight increase in the optical activity effect is observed, standing 1.4 fold higher compared to free naringin for the SPD derivative. The effect for the longer polyamine chain linkers, bearing more NH groups, is quite different, with quenching emerging, possibly due to induced rigidity brought about through bilateral flavonoid conjugation.

Naringenin-based derivatives exhibit a different fluorescence profile, with the EDA and TMEDA linkers being competitive in providing enhancement of the emission compared to the free flavonoid. Specifically, the Naringenin-TMEDA-Naringenin Schiff base derivative shows ~5-fold higher activity, whereas the Naringenin-EDA-Naringenin species exhibits 4-fold higher activity. Compared to the chemical structure of TMEDA, the longer-chain linker PMEDA shows approximately the same fluorescence effect as the free flavonoid. What is important is that, in the naringenin derivative, the linker DA-2-PrOH appears to induce complete quenching of the emission, with the remaining diamines following suit in effecting partial quenching. The overall picture is that most of the materials with enhanced or quenched emission exhibit a noticeable shift (especially in the naringin-based derivatives) of the emission maxima, which derive basically from enhanced electron delocalization or cause slight perturbations in the conjugated π-system of the flavonoid [62,63]. The longer chain linkers, SPD and SPM diamines, exhibit larger red-shifts in naringenin flavonoid derivatives due to increased flavonoid π-conjugation.

The distinct fluorescence behavior of naringin and naringenin Schiff base derivatives can be rationalized by considering both structural and electronic differences. Naringin, bearing the bulky sugar moiety on its side, is characterized by restricted π–π stacking and enhanced intramolecular hydrogen bonding, which, upon derivatization, are reduced, thus leading to enhancement of emission. In contrast, naringenin, lacking the sugar group, possesses a more planar aglycone framework that already allows for greater flavonoid π–π interactions and electron delocalization. In this case, linkers that can introduce compact geometries can show enhanced fluorescence compared to others, projecting conformational freedom and additional non-radiative relaxation channels, thereby causing partial quenching effects or limiting the induced enhancement. Taken together, these observations demonstrate that the sugar moiety in naringin favors fluorescence enhancement upon modification. On the other hand, naringenin requires optimal linker geometry and electronic contribution to bear a similar effect, thus highlighting the delicate rigidity-flexibility balance, amine conjugation, and excited-state deactivation in flavonoid Schiff base systems.

### 3.3. Antibacterial Activity

The initially designed and subsequently synthesized derivatives of naringin and naringenin have been employed in antibacterial studies against Gram (+) and Gram (−) bacteria. For that, the disk diffusion method was chosen due to low solubility of the materials in aqueous media and/or mixtures with bacterial media under assay conditions. It is important to emphasize that, during incubation, the pellets were observed to become visibly wetted by the agar medium, thus indicating partial dissolution and diffusion of the material into the surrounding matrix. This behavior supports the notion that the observed zones of inhibition arise from diffusion-mediated antibacterial activity rather than purely surface-bound effects. For this reason, four bacterial strains have been used, including two Gram (+) (*S. aureus* and *B. cereus*) and two Gram (−) (*E. coli* and *X. campestris*) ones. From an initial point of view, it is observed that flavonoid derivatives are selective toward the *E. coli* strain, as they exhibit a significant antibacterial effect compared to free flavonoids. However, the way of action of this specific activity is different for the two types of Schiff base-modified flavonoids. Specifically, naringin-based derivatives show a well-defined antibacterial effect, which may be linked to the combination of the flavonoid with the appropriate linker in a unique molecule. In such a setting, 2.0–3.0 mg (7.1–11.0 mg/cm^2^) is needed for the materials to be efficient in killing *E. coli*. For the EDA-based (no effect derived from the neat materials has been observed) derivative, approximately twice as large an amount (5.0 mg or 17 mg/cm^2^) is needed to observe an antimicrobial effect. On the other hand, naringenin derivatives exhibit antimicrobial efficacy through a higher amount (10 mg or 36 mg/cm^2^) of material. Compared with the neat starting material, there is almost no effect for naringenin, but there is considerable inhibition from linkers in the range from 0.64 mg or 2.3 mg/cm^2^ to 9.1 mg or 33 mg/cm^2^. This means that the newly synthesized derivatives of naringenin show an antimicrobial effect, but it is lower than the linker itself.

Overall, the selective effect against *E. coli* is consistent with the notion that the synthesized flavonoid derivatives can pass through the cell membrane of Gram (−) bacteria, amounting to rapid and intense bactericidal activity. Regardless, however, of their ability to traverse cell membranes and reach into metabolic pathways, the individual metabolic systems among the various Gram (−) negative bacteria may not be the same, thereby differentiating their phenotype toward the specific flavonoid derivatives, as in *X. campestris* vs. *E. coli*. The most important fact is that naringin and naringenin-based derivatives are shown to possess antibacterial activity that flavonoids themselves do not possess against *E. coli*. Selectivity against *E. coli* is possibly due to [18,64] (a) hydrogen bonding interactions, owing to the presence of (poly)amine groups in the hybrid flavonoid species, which may promote electrostatic interactions with the negatively charged lipopolysaccharide (LPS) layer of Gram (−) bacteria, (b) the diamine linkers facilitating penetration through porin channels or promoting local disruption of the outer membrane, whereas long flexible polyamine linkers may favor membrane association yet also increase non-specific aggregation, (c) the imine groups being capable of indigenous metal chelation and hydrogen bonding interactions with proteins, potentially interfering with molecular internalization processes, and (d) strain-specific defense mechanisms (outer-membrane composition, efflux pumps, secretion systems, exopolysaccharide layers) that may render *S. aureus*, *B. cereus* and *X. campestris* less susceptible to membrane integrity compromise under the present conditions. The aforementioned contentions may be further rationalized through membrane-permeability assays, propidium iodide uptake, and ROS measurements.

Collectively, the experimental results emerging from optical and antibacterial work on the synthesized Schiff base derivatives highlight the strong interconnection and relation between electronic structure, molecular rigidity, and biological interactions. In the case of naringin-based derivatives, the sugar moiety likely introduces steric hindrance and hydrogen-bond networks that suppress non-radiative decay, which, upon Schiff base condensation, are converted into radiative pathways, thereby producing higher fluorescence. Consequently, the herein provided plausible interpretations of the observed effects are consistent with the experimental trends and may involve steric effects, hydrogen bonding, and molecular packing. Further validation, however, is necessary through crystallographic or computational analysis. The same derivatized molecules are shown to have increased hydrophilicity and metal/DNA chelation ability, thus enabling selective penetration of the *E. coli* strain membrane structure. On the other hand, naringenin-based derivatives (bearing no bulky sugar groups) seem to display their dual activity role(s), relying more on the electronic contribution originating in the linkers used. This explains why only compact electron-donating linkers, like EDA and TMEDA, enhance fluorescence. The combination of fluorescence and antibacterial properties suggests that structural modifications, providing excited-state stabilization and fluorescence enhancement, are closely related to those driving bacterial inhibition. In that respect, new flavonoid-based multifunctional materials emerge as promising agents for antimicrobial and sensing applications.

## 4. Materials and Methods

### 4.1. Flavonoids and Chemicals

All reactions and manipulations were carried out under aerobic conditions. The following starting materials were used without further purification. Flavonoids, naringin monohydrate (C_27_H_32_O_14_·H_2_O) and naringenin (C_15_H_12_O_5_), were purchased from Sigma-Aldrich (Sigma-Aldrich Chemical Company, Steinheim, Germany) and BLD Pharm (BLD Pharmtech Ltd., Reinbek, Germany), respectively. Diamines and polyamines were supplied as follows: Ethylenediamine (NH_2_(CH_2_)_2_NH_2_, EDA) from BDH (BDH Chemicals, Dorset, UK), 1,3-diamino-2-propanol (NH_2_CH_2_CH(OH)CH_2_NH_2_, DA-2-PrOH) from J&K (J&K Scientific GmbH, Marbach, Germany), Tetramethylenediamine (NH_2_(CH_2_)_4_NH_2_, TMEDA), Pentamethylenediamine (NH_2_(CH_2_)_5_NH_2_, PMEDA), Spermidine (NH_2_(CH_2_)_3_NH(CH_2_)_4_NH_2_, SPD), and Spermine dihydrate (NH_2_(CH_2_)_3_NH(CH_2_)_4_NH(CH_2_)_3_NH_2_·2H_2_O, SPM) from Alfa Aesar (Alfa Aesar, Karlsruhe, Germany). Ethanol, Methanol LC-MS grade and dimethyl sulfoxide (DMSO) were supplied by Sigma-Aldrich (Sigma-Aldrich Chemical Company, Steinheim, Germany).

### 4.2. Physical Measurements

A ThermoFinnigan Flash EA 1112 CHNS elemental analyzer (Thermo Fisher Scientific Inc., Waltham, MA, USA) was used for quantitative determination of carbon, hydrogen, and nitrogen. Operation of the analyzer relies on the dynamic flash combustion of samples at 1800 °C. Determination of the products was further pursued through reduction, trapping, complete GC separation, and detection in a fully automated manner and controlled by PC through the Eager 300 (v 1.0) dedicated software system.

A Nicolet FT-IR 200 spectrometer (Thermo Fisher Scientific Inc., USA) was used to collect FT-Infrared spectra, using KBr pellets, at room temperature conditions, and 64 scans.

UV-Visible spectroscopy measurements of all solutions, prepared in DMSO, were carried out on a Hitachi U-1900 spectrophotometer (Hitachi Ltd., Tokyo, Japan), in the range 200–600 nm, through serial dilutions in the 10^−3^ to 10^−5^ M concentration range.

#### 4.2.1. ESI-MS Spectrometry

Electrospray ionization mass spectrometry (ESI-MS) (Thermo Scientific, Bremen, Germany) on a Thermo Fisher Scientific model LTQ Orbitrap Discovery mass spectrometer was employed for mass spectrometric measurements of all investigated materials in methanolic solutions, at a concentration 10^−4^ M. All solutions of the modified flavonoids with molecular formula: M1 = Naringin-EDA-Naringin = C_56_H_68_N_2_O_26_; M2 = Naringin-DA-2-PrOH-Naringin = C_57_H_70_N_2_O_27_; M3 = Naringin-TMEDA-Naringin = C_58_H_72_N_2_O_26_; M4 = Naringin-PMEDA-Naringin = C_59_H_74_N_2_O_26_; M5 = Naringin-SPD-Naringin = C_61_H_79_N_3_O_26_; M6 = Naringin-SPM-Naringin = C_64_H_86_N_4_O_26_; M7 = Naringenin-EDA-Naringenin = C_32_H_28_N_2_O_8_; M8 = Naringenin-DA-2-PrOH-Naringenin = C_33_H_30_N_2_O_9_; M9 = Naringenin-TMEDA-Naringenin = C_34_H_32_N_2_O_8_; M10 = Naringenin-PMEDA-Naringenin = C_35_H_34_N_2_O_8_; M11 = Naringenin-SPD-Naringenin = C_37_H_39_N_3_O_8_; and M12 = Naringenin-SPM-Naringenin = C_40_H_46_N_4_O_8_; were introduced to the ESI source, operating in a positive ionization mode, with a flow rate of 5 μL/min, using an integrated syringe pump. Source operating conditions were: 3.7 kV spray voltage, and 320 °C heated capillary temperature.

#### 4.2.2. Solution ^1^H-, ^13^C-NMR

One-dimensional Nuclear Magnetic Resonance Spectroscopy experiments were carried out on a Varian 600 MHz spectrometer (Agilent, Germany), using DMSO-d^6^ (Sigma-Aldrich Chemical Company, Steinheim, Germany) at 298 K. An internal standard, 3-(Trimethylsilyl)-propionic-2,2,3,3-d^4^ acid sodium salt (TSP) (Sigma-Aldrich Chemical Company, Steinheim, Germany), was used in every experiment. Proton (^1^H) spectra were acquired with 512 transients and a spectral width of 5000 Hz, while ^1^H PRESAT was conducted using eight scans and a relaxation delay of 2 s through a two-step purge experiment. Carbon (^13^C) spectra were acquired with 5000 transients, a spectral width of 37,000 Hz, and a relaxation delay of 5 s. Experimental data were processed using VNMR routines, and all spectra were zero-filled and subjected to exponential apodization prior to Fourier Transformation (FT). Chemical shifts (δ) are reported in ppm and the overall spectra were analyzed through the Mnova 6.0.2 software (Mestrelab Research S.L., Santiago de Compostela, Spain).

#### 4.2.3. Photoluminescence

The solid-state photoluminescence activity (emission (em) and excitation (ex)) of all materials was investigated on a Hitachi F-7000 fluorescence spectrophotometer from Hitachi High-Technologies Corporation (Hitachi Ltd., Japan). The hardware was supported by Windows XP, and the system employed the FL Solutions 2.1 software. All solid-state spectra were recorded using split widths of 5.0 nm and a scan speed of 1200 nm min^−1^ at room temperature conditions.

### 4.3. Synthesis of Schiff Base Modified Flavonoids

#### General Procedure

Solid flavonoid (naringin or naringenin) and the selected diamine or polyamine linkers, dissolved in ethanol solution, were mixed in a molar ratio 2:1. Typically, 0.58 g (1.0 mmol) of naringin (or the equivalent molar amount of naringenin (0.27 g)) was placed in a 50 mL round-bottom flask. In a test tube, 10 mL of ethanol and 33 μL (0.50 mmol) of EDA (or the appropriate diamine/polyamine) were mixed via vigorous vortexing. The diamine/polyamine solution was slowly added to the flavonoid solution under continuous stirring. The reaction mixture was heated under reflux for 24 h or transferred to a Teflon-lined stainless-steel reactor (23 mL) and heated at 100 °C for 24 h (in the case of the reaction between naringenin and DA-2-PrOH). After completion of the reaction, the final reaction mixture was isolated by filtration and dried in open air.

**Note:** The same general procedure was applied for all compounds listed in Table 4 for naringin derivatives and in Table 5 for naringenin derivatives, with the amounts of diamine/polyamine adjusted accordingly to the molecular weight in order to maintain the 2:1 flavonoid/linker molar ratio. Analytical calculations and yields are also listed in Table 4 and Table 5.

### 4.4. Antibacterial Properties In Vitro

Gram positive (Gram (+)) (*Staphylococcus aureus*; *S. aureus*, and *Bacillus cereus*; *B. cereus*) and Gram negative (Gram (−)) (*Escherichia coli*; *E. coli*, and *Xanthomonas campestris*; *X. campestris*) bacterial cultures were grown on autoclavable 25 mL Luria–Bertani agar (LB agar) (Applichem PanReac, Darmstadt, Germany) Petri dishes with a diameter of 90 mm [65]. To that end, solid state materials were employed in the experiments and investigated using the disk diffusion method. In that respect, pellet samples (6 mm in diameter) were prepared, containing the studied material and LB Agar mixtures at different concentrations, with a total testing material mass of 30 mg. Minimum Inhibitory Concentration (MIC) (expressed as mass amount (mg) and mg/cm^2^ (mg/area of studied disk = mg/0.28 cm^2^)) values were determined though appropriate measurement of standard Zone Of Inhibition (ZOI), following incubation for 12–15 h at 37 °C (or 30 °C for *X. campestris*) with bacteria and tested samples [66]. More specifically, bacteria were grown in 3 mL LB broth, using a 25 mL Erlenmeyer flask in an Edmund Bühler TH15 shaking incubator (Edmund Bühler GmbH, Bodelshausen, Germany) at 37 °C for 1–2 h (or 30 °C for 4 h in case of *X. campestris*), until the O.D. at 600 nm reached a value of 0.5 (or 1.2 for *X. campestris*), corresponding to the exponential phase of the growth curve (5 × 10^5^ CFU·mL^−1^). Subsequently, optical density (O.D.) measurements were carried out on a Hitachi UV-Visible U-2800 spectrophotometer (Hitachi, Tokyo, Japan). All experiments were run in triplicate under aseptic conditions. The Luria–Bertani broth (LB broth) (Sigma Aldrich, Munich, Germany) and penicillin–streptomycin (Biowest, Nuaillé, France) were used as negative and positive control, respectively.

### 4.5. Statistical Analysis

All obtained experimental data were collected in triplicate and further presented as average ± SD values of multiple sets of independent measurements. All statistical analyses were performed using GraphPad Prism v.6 (GraphPad Software Inc., Boston, MA, USA).

## 5. Conclusions

The presented research activity highlights successfully designed and synthetically pursued new hybrid derivatives of naringin and naringenin through Schiff base condensation reactions, using diamine and polyamine linkers. The implemented idea makes use of two different types of natural organic agents (flavonoids and (poly)amines) and combines them into a single ternary molecule, involving two flavonoid molecules and one appended linker (acting as a bridge). The emerging model reflects a new molecular entity exemplifying a higher concentration of flavonoids in a single molecule that could exhibit differentiated and hopefully uniquely defined biological activity profile(s). The molecular model employed in this endeavor makes use of two intimately related flavonoids, naringin and naringenin, with the presence/absence of a differentially appended disaccharide on one of them, serving as a control to their (bio)chemical reactivity and physicochemical properties. The attempted structural modification of the flavonoids, via introduction of the imine (–C=N–) moiety, provided extended conjugation and altered charge-transfer characteristics, resulting in significant changes in their optical behavior. Naringin-based derivatives generally exhibited stronger fluorescence properties through enhancement of emission bands, with naringenin-based systems exhibiting more selective improvements, depending on the electronic nature of the linker. The physicochemically characterized ternary materials revealed properties that support both optical and potentially unique biological activity attributes. Consequently, antibacterial studies were further pursued under specific conditions, revealing selective and pronounced activity against *E. coli*, thereby demonstrating that Schiff base modification not only enhances the intrinsic physicochemical properties of flavonoids but also introduces a new biological selectivity attribute not present in the free flavonoids. Collectively, both physical and (bio)chemical findings highlight the potential of Schiff base flavonoid derivatives as multifunctional agents, which are capable of exhibiting bioactivity and fluorescence. To that end, the new materials set the basis for further perusal of their (bio)chemical reactivity toward metal ion coordination, sensing, detection, and catalytic applications, thereby opening up the field of molecular engineering hybrid “dense” natural products (vide supra) and seeking new opportunities in sensing applications and antimicrobial agents. Development of such custom-designed and synthetically pursued (bi)multifunctional materials is currently under investigation in our lab.

## Figures and Tables

**Table 1:** FT-IR vibrations and UV-Visible spectral data of free and Schiff base modified flavonoids.

Flavonoid/Modified Flavonoid	FT-IR Vibrations (cm^−1^)				UV-Visible	
	OH	C-H	C=N	C-O-C	Wavelength (λ_max_, nm)	Molecular Absorption Coefficient (ε, M^−1^·cm^−1^)
Naringin	3417	2926	1649(C=O)	1205–1043	284/320	16,579/3215
Naringin-EDA-Naringin	3375	2973	1612	1179–1064	284/392	18,780/3694
Naringin-DA-2-PrOH-Naringin	3382	2978	1607	1179–1064	286/392	18,825/5299
Naringin-TMEDA-Naringin	3406	2935	1611	1177–1065	286/389	18,535/5121
Naringin-PMEDA-Naringin	3396	2930	1611	1167–1075	286/389	18,657/5692
Naringin-SPD-Naringin	3406	2933	1613	1180–1066	285/386	25,517/6075
Naringin-SPM-Naringin	3402	2933	1608	1180–1070	285/386	24,469/5640
Naringenin	3285/3118	2911	1633(C=O)	1161–1065	288/324	18,412/3495
Naringenin-EDA-Naringenin	3369/3192	3032	1589	1188–1010	286/386	26,671/7080
Naringenin-DA-2-PrOH-Naringenin	3375/3187	2916	1596	1184–1012	297/386	18,950/5158
Naringenin-TMEDA-Naringenin	3422/3171	2967	1590	1177–1069	294/380	18,510/6284
Naringenin-PMEDA-Naringenin	3418/3154	2941	1589	1177–1021	297/386	18,327/5942
Naringenin-SPD-Naringenin	3427/3214	2927	1592	1170–1060	303/387	18,535/5756
Naringenin-SPM-Naringenin	3402/3211	2930	1592	1171–1060	288/385	4987/1063

**Table 2:** ZOI values for naringin Schiff base derivatives in Gram (−) (*E. coli*) bacterium.

Derivatives	Mass Content (mg)/Mass Content/Unit Area (mg/cm^2^) ZOI (mm)	Controls	Mass Content (mg)/Mass Content/Unit Area (mg/cm^2^) ZOI (mm)
Naringin-EDA-Naringin	5.0 mg (17 mg/cm^2^) 28.2 ± 0.1	Naringin	4.9 mg (17 mg/cm^2^) n.e.z.
		EDA	0.23 mg (0.82 mg/cm^2^) n.e.z.
Naringin-DA-2-PrOH-Naringin	2.0 mg (7.1 mg/cm^2^) 26.0 ± 0.2	Naringin	1.9 mg (6.8 mg/cm^2^) n.e.z.
		DA-2-PrOH	0.35 mg (1.3 mg/cm^2^) n.e.z.
Naringin-TMEDA-Naringin	2.0 mg (7.1 mg/cm^2^) 38.3 ± 0.3	Naringin	1.9 mg (6.8 mg/cm^2^) n.e.z.
		TMEDA	0.14 mg (0.5 mg/cm^2^) n.e.z.
Naringin-PMEDA-Naringin	2.0 mg (7.1 mg/cm^2^) 29.8 ± 0.2	Naringin	1.9 mg (6.8 mg/cm^2^) n.e.z.
		PMEDA	0.16 mg (0.57 mg/cm^2^) n.e.z.
Naringin-SPD-Naringin	3.0 mg (11 mg/cm^2^) 26.6 ± 0.2	Naringin	2.7 mg (9.6 mg/cm^2^) n.e.z.
		SPD	0.33 mg (1.2 mg/cm^2^) n.e.z.
Naringin-SPM-Naringin	3.0 mg (11 mg/cm^2^) 25.5 ± 0.2	Naringin	2.6 mg (9.3 mg/cm^2^) n.e.z.
		SPM	0.45 mg (1.6 mg/cm^2^) n.e.z.

**Note:** n.e.z.: no evaluable zone. 1% Penicillin/Streptomycin in *E. coli* show ZOI of 19.8 ± 0.2 mm.

**Table 3:** ZOI values for naringenin Schiff base derivatives in Gram (−) (*E. coli*) bacterium.

Derivatives	Mass Content (mg)/Mass Content/Unit Area (mg/cm^2^) ZOI (mm)	Controls	Mass Content (mg)/Mass Content/Unit Area (mg/cm^2^) ZOI (mm)
Naringenin-EDA-Naringenin	10 mg (36 mg/cm^2^) 23.3 ± 0.2	Naringenin	9.5 mg (34 mg/cm^2^) n.e.z.
		Ethylenediamine	0.9 mg (3.2 mg/cm^2^) 13.2 ± 0.1
Naringenin-DA-2-PrOH-Naringenin	10 mg (36 mg/cm^2^) 17.3 ± 0.1	Naringenin	9.0 mg (32 mg/cm^2^) n.e.z.
		1,3-diamino-2-propanol	1.4 mg (5.0 mg/cm^2^) 11.1 ± 0.1
Naringenin-TMEDA-Naringenin	10 mg (36 mg/cm^2^) 19.9 ± 0.1	Naringenin	9.1 mg (33 mg/cm^2^) n.e.z.
		1,4-diaminobutane	1.4 mg (5.0 mg/cm^2^) 18.2 ± 0.1
Naringenin-PMEDA-Naringenin	10 mg (36 mg/cm^2^) 16.5 ± 0.3	Naringenin	1.9 mg (6.8 mg/cm^2^) n.e.z.
		1,5-diaminopentane	1.6 mg (5.7 mg/cm^2^) 18.4 ± 0.1
Naringenin-SPD-Naringenin	10 mg (36 mg/cm^2^) 26.9 ± 0.1	Naringenin	8.3 mg (30 mg/cm^2^) n.e.z.
		Spermidine	0.64 mg (2.3 mg/cm^2^) 16.2 ± 0.2
Naringenin-SPM-Naringenin	10 mg (36 mg/cm^2^) 21.5 ± 0.1	Naringenin	7.5 mg (27 mg/cm^2^) n.e.z.
		Spermine	2.8 mg (10 mg/cm^2^) 34.4 ± 0.3

**Note:** n.e.z.: no evaluable zone. 1% Penicillin/Streptomycin in *E. coli* shows a ZOI of 19.8 ± 0.2 mm.

**Table 4:** Synthetic and analytical details of naringin derivatives, using 0.58 g (1.0 mmol) naringin.

Linker Mass 0.50 mmol	Chemical Formula	MW (g/mol)	Product (g) Yield (%)	% C Calc./Found	% H Calc./Found	% N Calc./Found
33 μL EDA	C_56_H_68_N_2_O_26_	1185.14	0.37 g 63%	56.71/56.75	5.74/5.76	2.36/2.33
0.045 g DA-2-PrOH	C_57_H_70_N_2_O_27_	1215.16	0.23 g 38%	56.29/56.22	5.76/5.78	2.30/2.28
0.045 g TMEDA	C_58_H_72_N_2_O_26_	1213.19	0.36 g 59%	57.36/57.41	5.93/5.96	2.31/2.27
0.051 g PMEDA	C_59_H_74_N_2_O_26_	1227.21	0.59 g 95%	57.69/57.62	6.03/6.06	2.28/2.24
78 μL SPD	C_61_H_79_N_3_O_26_	1270.28	0.15 g 52%	57.62/57.64	6.22/6.26	3.31/2.28
0.12 g SPM	C_64_H_86_N_4_O_26_	1327.38	0.25 g 75%	57.86/57.81	6.48/6.42	4.22/4.25

**Table 5:** Synthesis details and elemental analysis of naringenin modified flavonoids, using 0.27 g (1.0 mmol) naringenin.

Linker Mass 0.50 mmol	Chemical Formula	MW (g/mol)	Product (g) Yield (%)	% C Calc./Found	% H Calc./Found	% N Calc./Found
33 μL EDA	C_32_H_28_N_2_O_8_	568.57	0.070 g 25%	67.58/67.60	4.93/4.96	4.93/4.90
0.045 g DA-2-PrOH	C_33_H_30_N_2_O_9_	598.60	0.14 g 47%	66.20/66.17	5.02/5.06	4.68/4.66
0.045 g TMEDA	C_34_H_32_N_2_O_8_	596.63	0.080 g 27%	68.38/68.41	5.36/5.40	4.69/4.67
0.051 g PMEDA	C_35_H_34_N_2_O_8_	610.65	0.13 g 43%	68.78/68.82	5.57/5.61	4.59/4.60
78 μL SPD	C_37_H_39_N_3_O_8_	653.72	0.12 g 36%	67.91/67.95	5.97/6.01	6.42/6.45
0.12 g SPM	C_40_H_46_N_4_O_8_	710.82	0.11 g 31%	67.52/67.46	6.47/6.41	7.88/7.85

## Data Availability

The original contributions presented in this study are included in the article/Supplementary Materials. Further inquiries can be directed to the corresponding authors.

## Supplementary Materials

The supporting information can be downloaded at: https://www.mdpi.com/article/10.3390/ijms27010253/s1.

## Abbreviations

The following abbreviations are used in this manuscript:

DA-2-PrOH	1,3-diamino-2-propanol
DMSO	Dimethyl sulfoxide
ESI-MS	Electron Spray Ionization Mass Spectrometry
EDA	Ethylenediamine
FT-IR	Fourier Transform Infrared Spectroscopy
MIC	Minimum Inhibitory Concentration
NMR	Nuclear Magnetic Resonance Spectroscopy
PMEDA	Pentamethylenediamine
PRESAT	Presaturation
SD	Standard deviation
SPD	Spermidine
SPM	Spermine
UV-Visible	Ultraviolet-Visible Spectroscopy
TMEDA	Tetramethylenediamine
ZOI	Zone Of Inhibition
