# Supplementary material for: Modified Flavonoids with Diamines and Polyamines Provide Enhanced Fluorescence and Antimicrobial Activity
Source: Int J Mol Sci. 2025 Dec 25;27(1):253. doi: 10.3390/ijms27010253 (PMC12785322; doi:10.3390/ijms27010253)
Supplement: Supplementary file 1 [file ijms-27-00253-s001.zip › ijms-4004357-supplementary.pdf]

Supplementary Information

Modified flavonoids with diamines and polyamines provide enhanced fluorescence and antimicrobial activity

Sevasti Matsia * and Athanasios Salifoglou *

Laboratory of Inorganic Chemistry and Advanced Materials, School of Chemical Engineering, Aristotle University of Thessaloniki, 54124 Thessaloniki, Greece

* Correspondence: srmatsia@auth.gr (S.M.); salif@auth.gr (A.S.); Tel.: +30-2310-996-204 (S.M.); +30-2310-996-179 (A.S.)

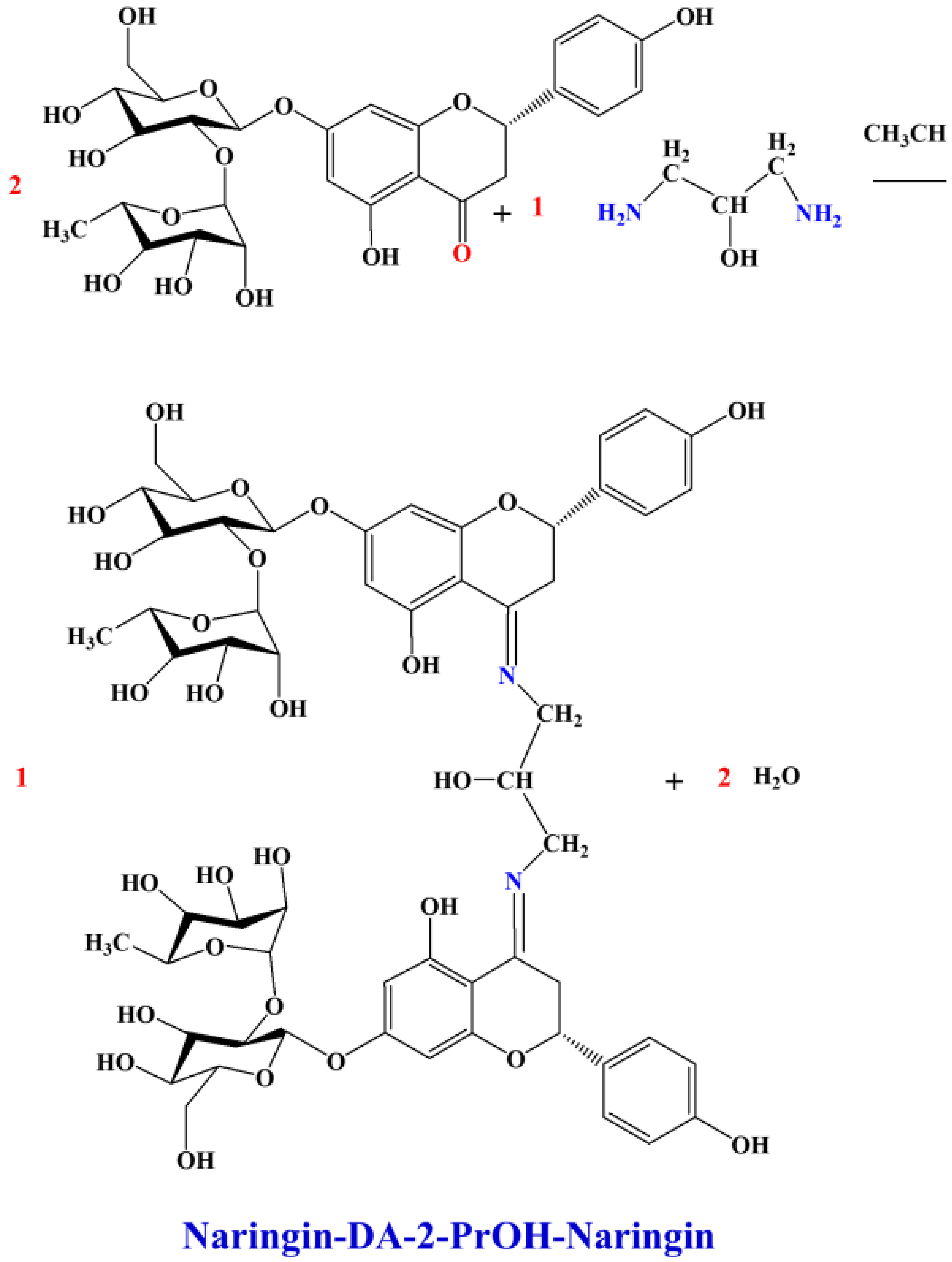

Reactivity Scheme S1. Naringin Schiff base reactivity reaction with DA-2-PrOH

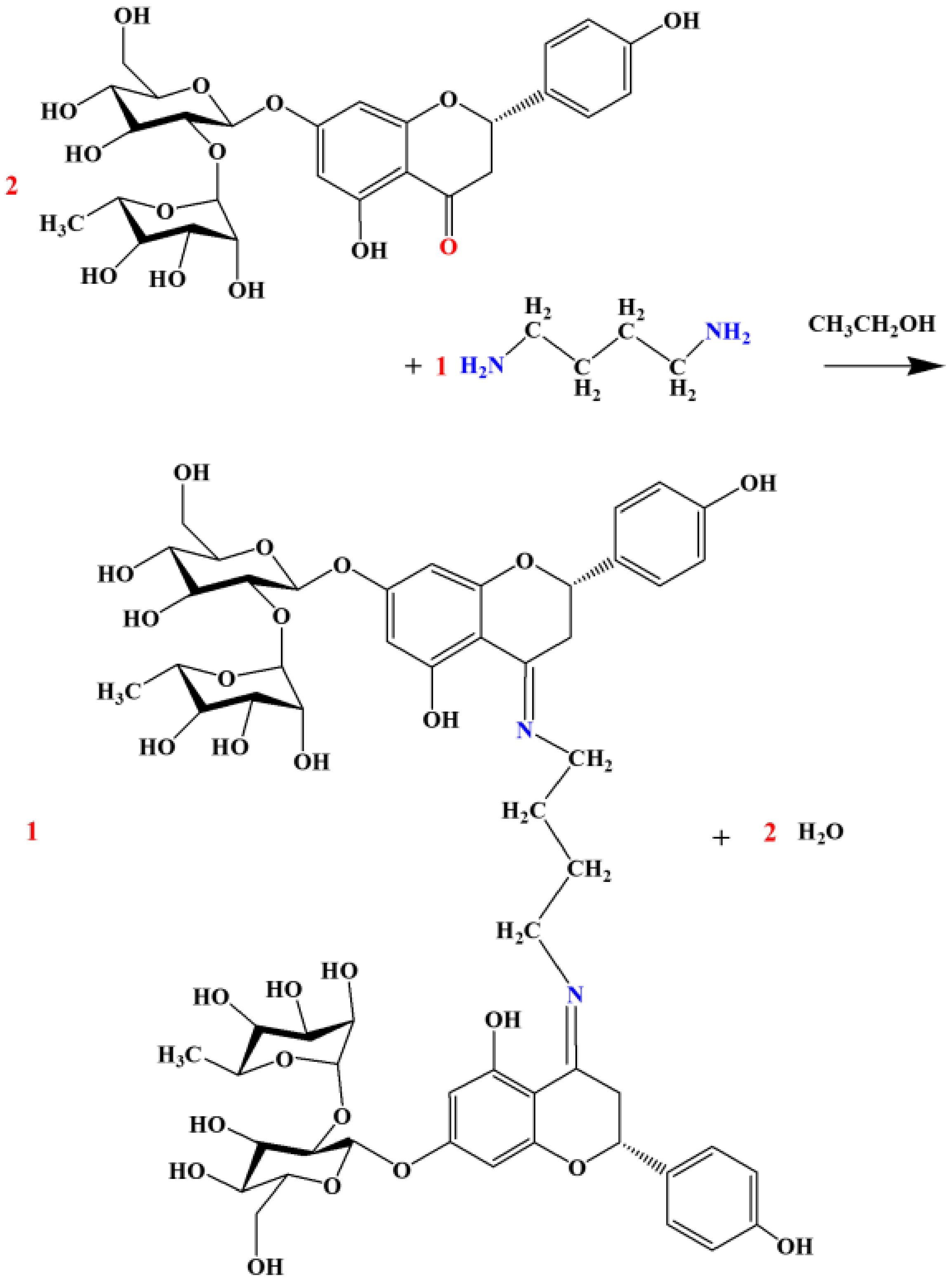

Naringin-TMEDA-Naringin

Reactivity Scheme S2. Naringin Schiff base reactivity reaction with TMEDA

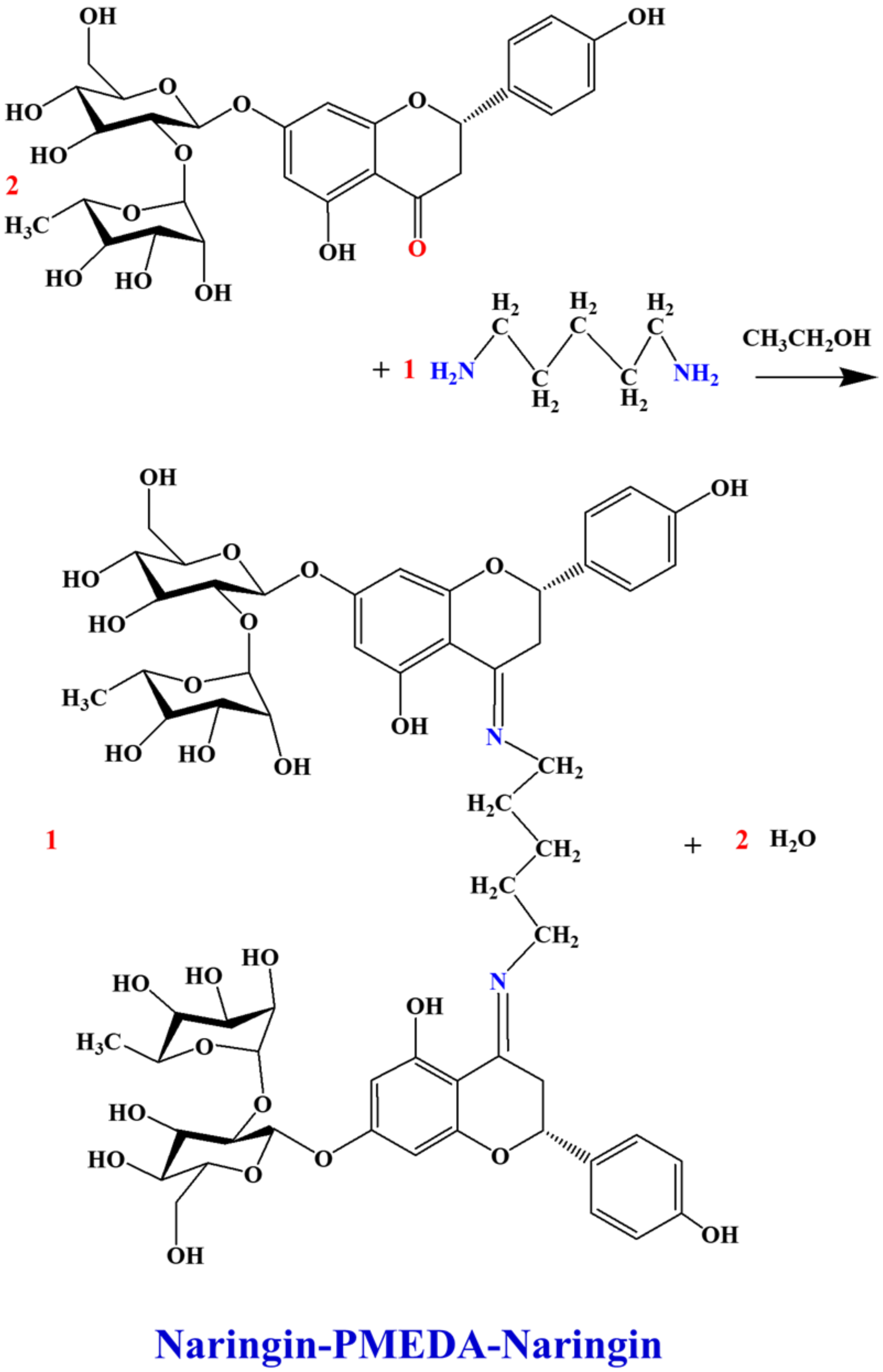

Reactivity Scheme S3. Naringin Schiff base reactivity reaction with PMEDA

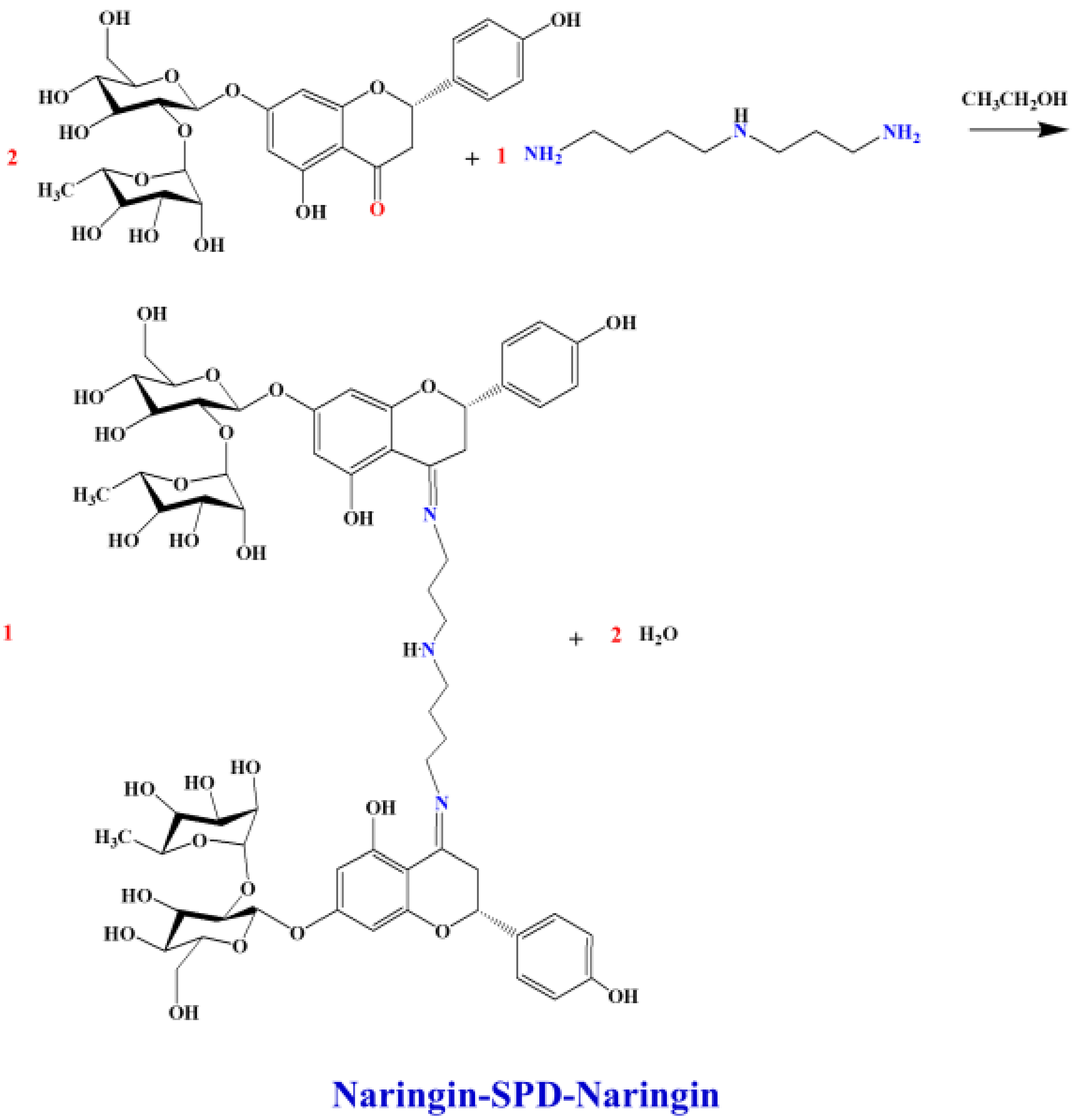

Reactivity Scheme S4. Naringin Schiff base reactivity reaction with SPD

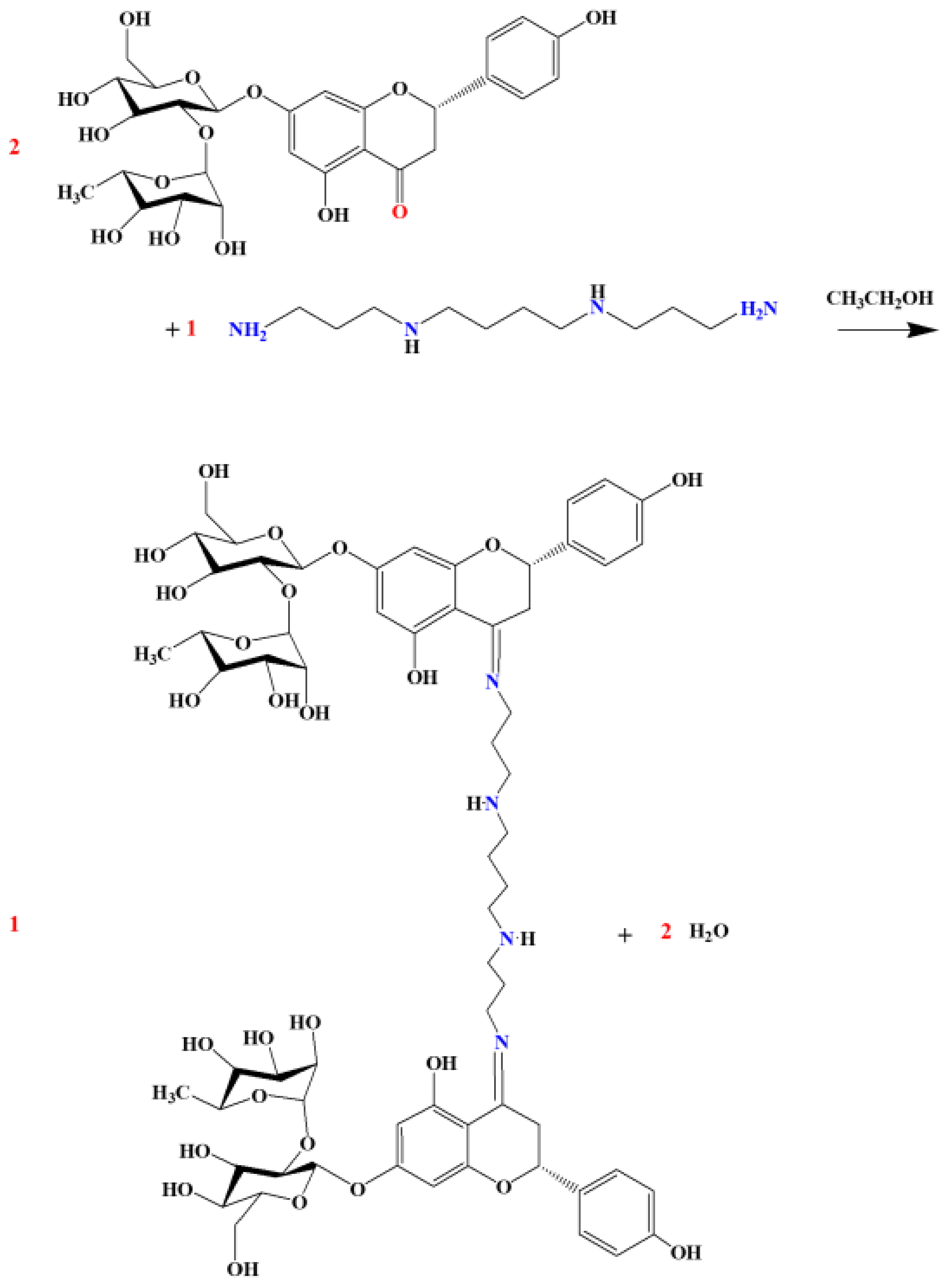

Naringin-SPM-Naringin

Reactivity Scheme S5. Naringin Schiff base reactivity reaction with SPM

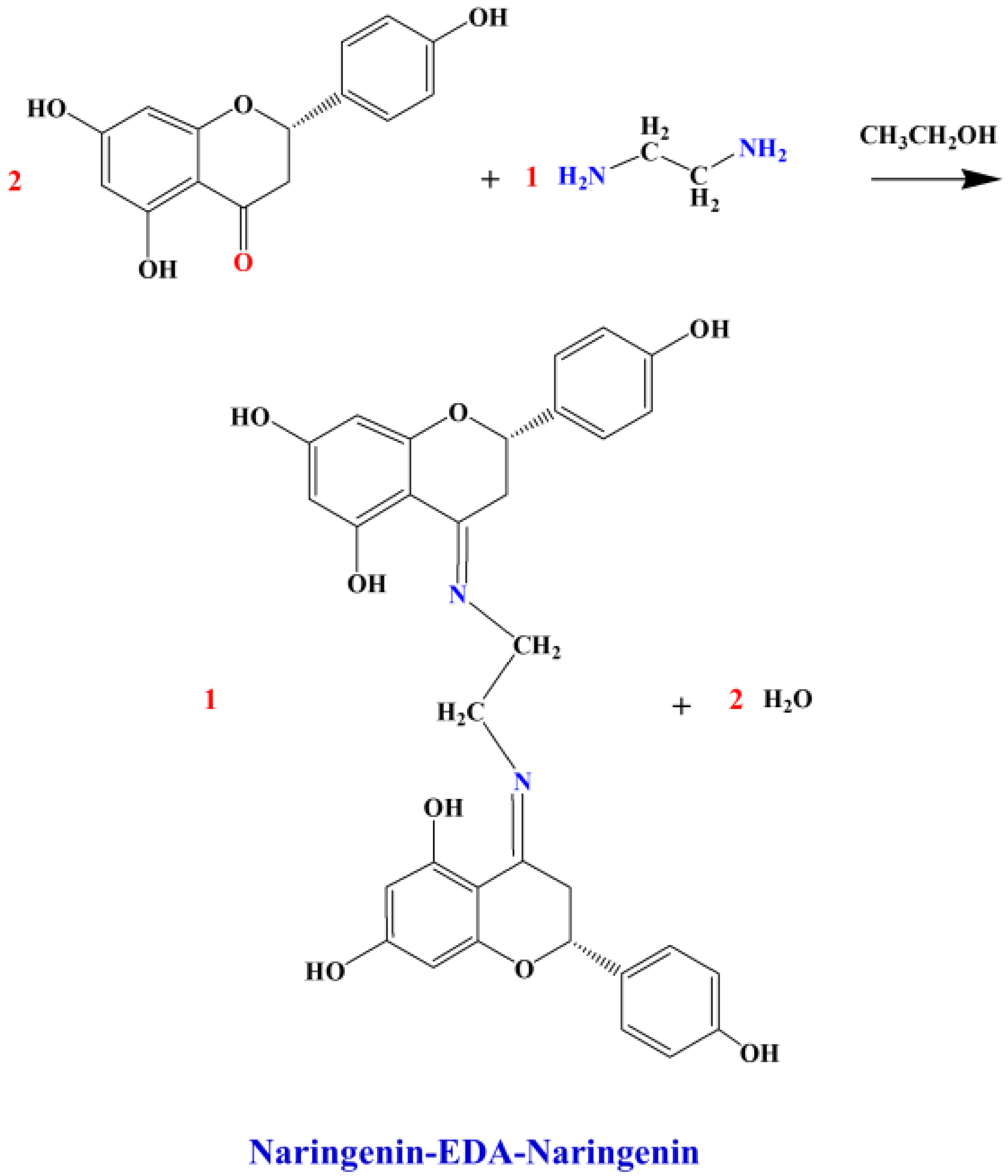

Reactivity Scheme S6. Naringenin Schiff base reactivity reaction with EDA

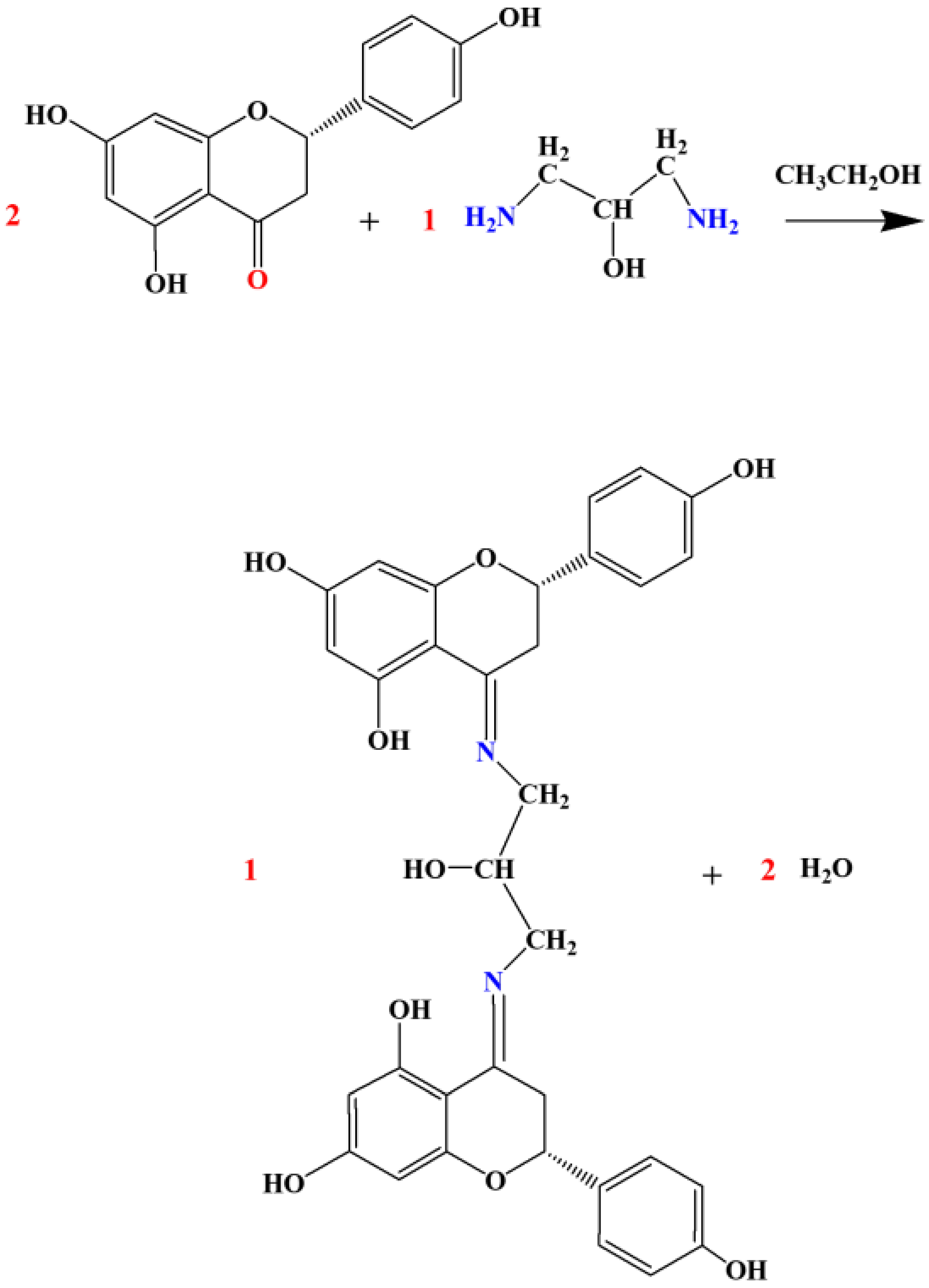

Naringenin-DA-2-PrOH-Naringenin

Reactivity Scheme S7. Naringenin Schiff base reactivity reaction with DA-2-PrOH

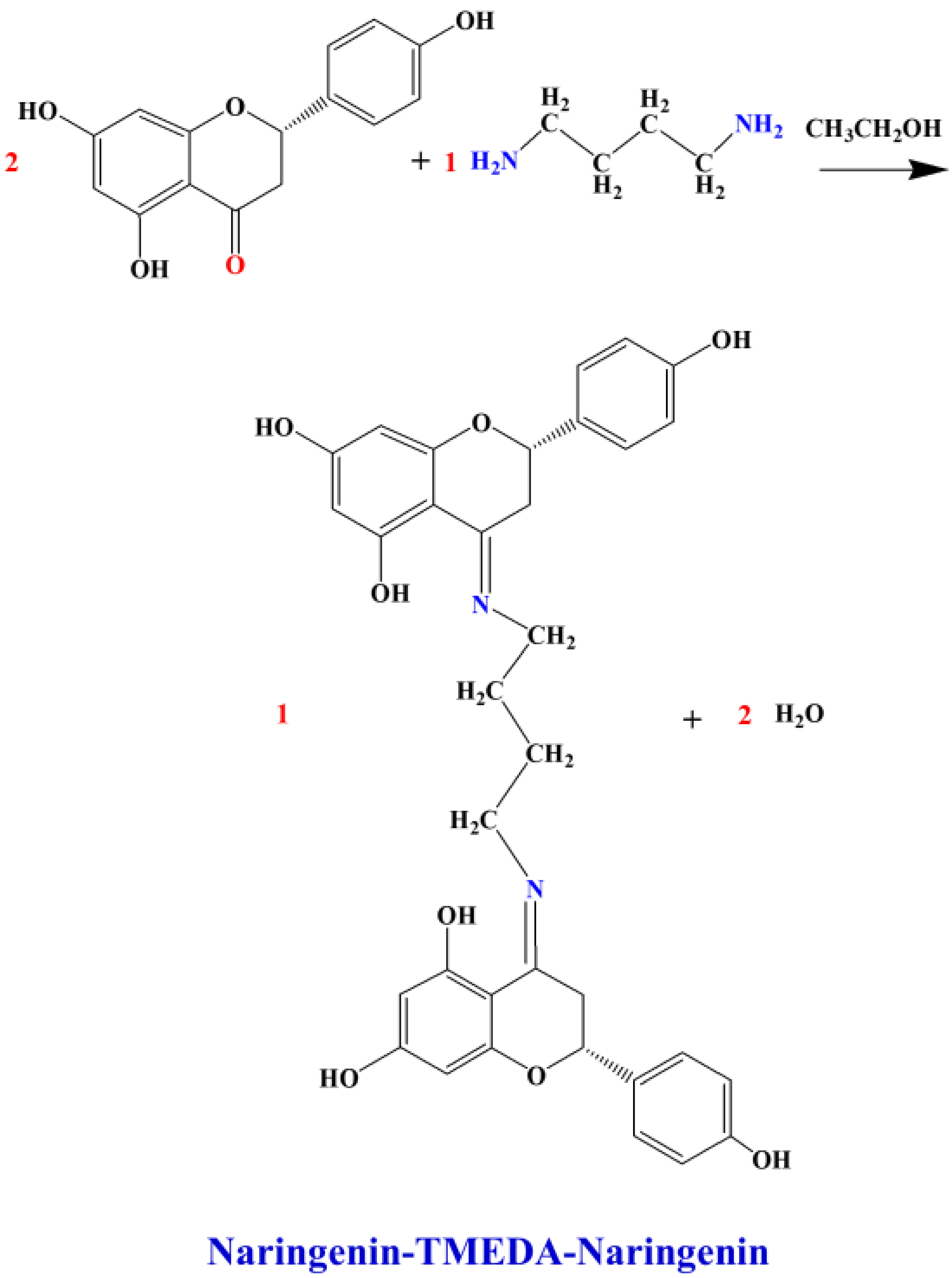

Reactivity Scheme S8. Naringenin Schiff base reactivity reaction with TMEDA

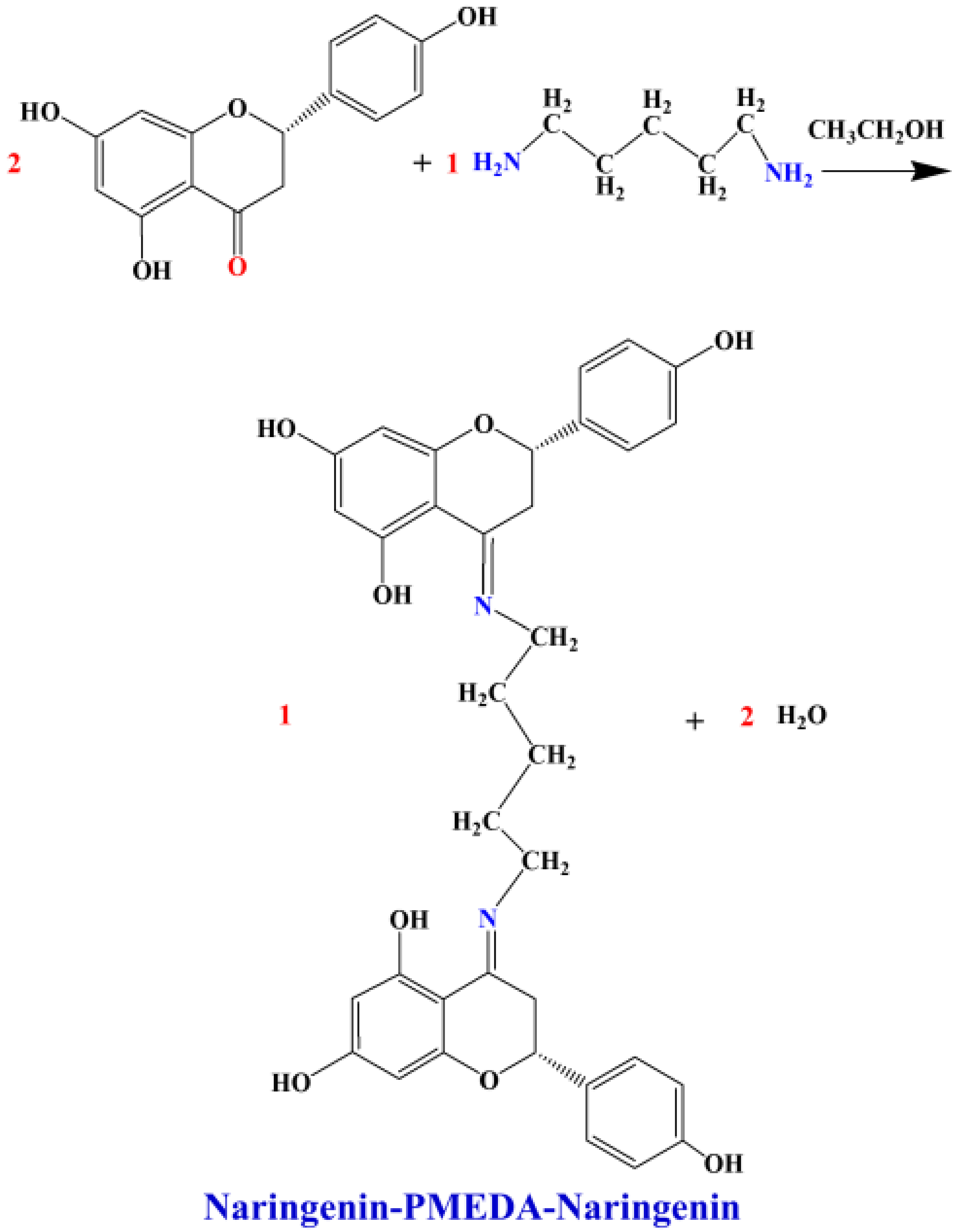

Reactivity Scheme S9. Naringenin Schiff base reactivity reaction with PMEDA

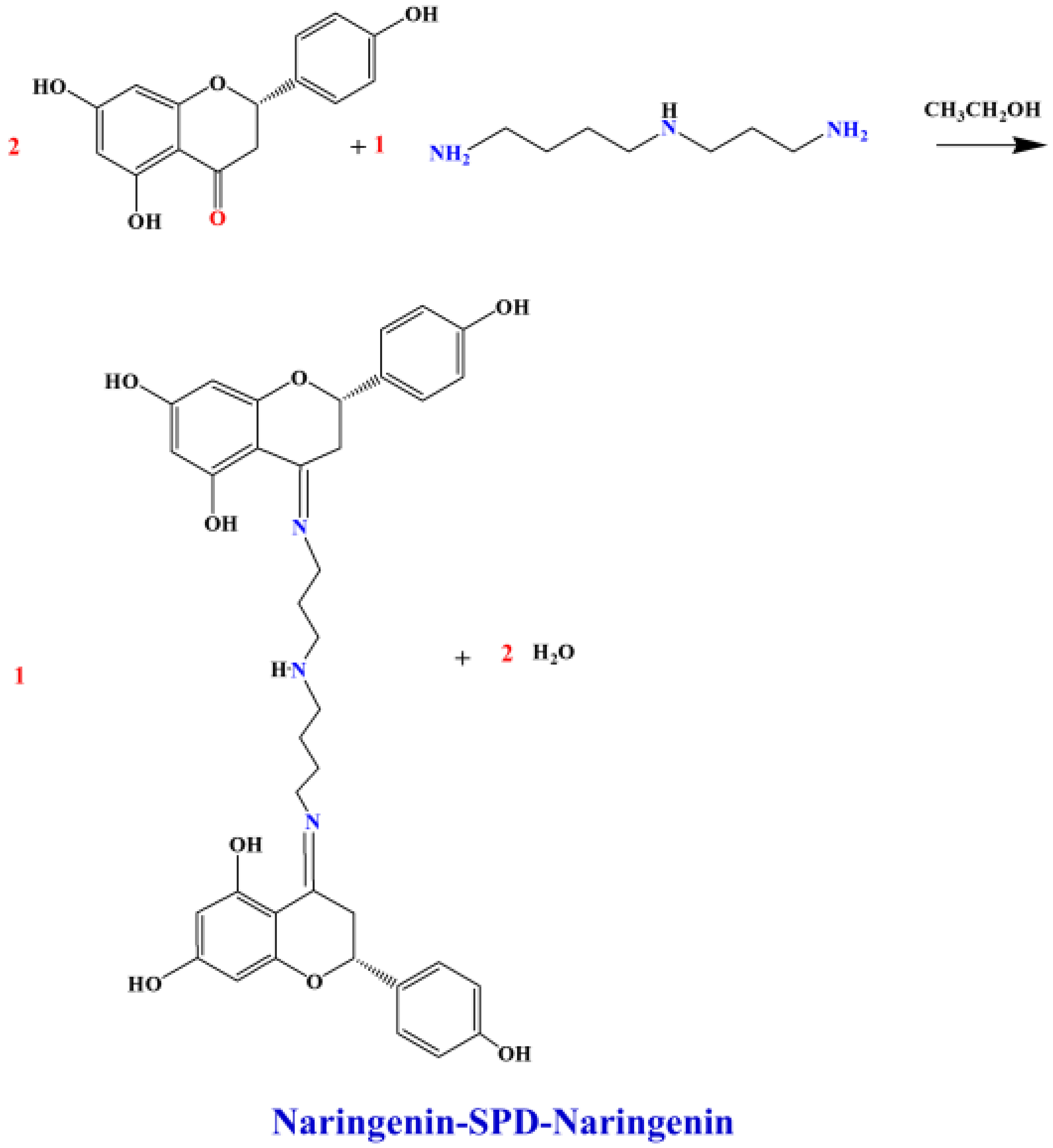

Reactivity Scheme S10. Naringenin Schiff base reactivity reaction with SPD

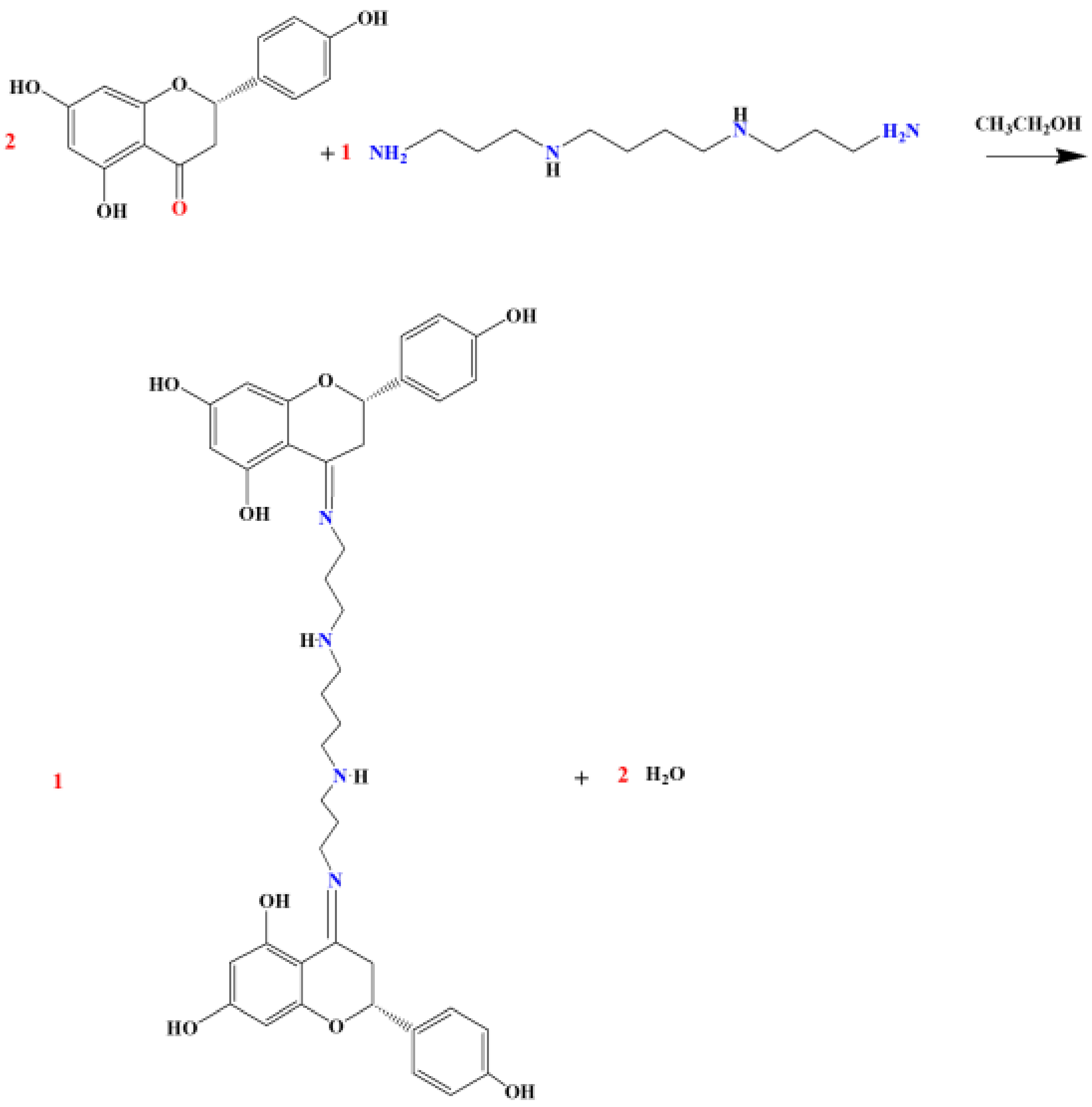

Naringenin-SPM-Naringenin

Reactivity Scheme S11. Naringenin Schiff base reactivity reaction with SPM

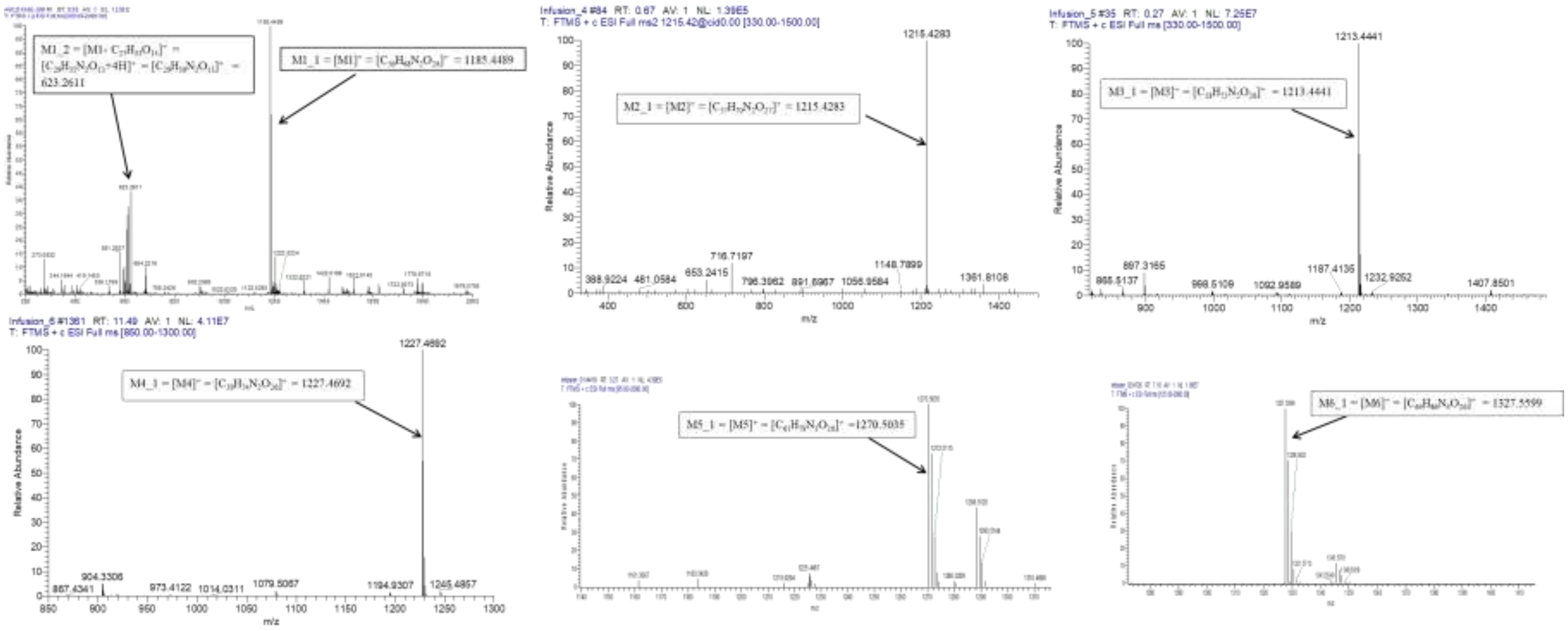

Figure S1. ESI-MS spectra of Naringin derivatives recorded in positive mode

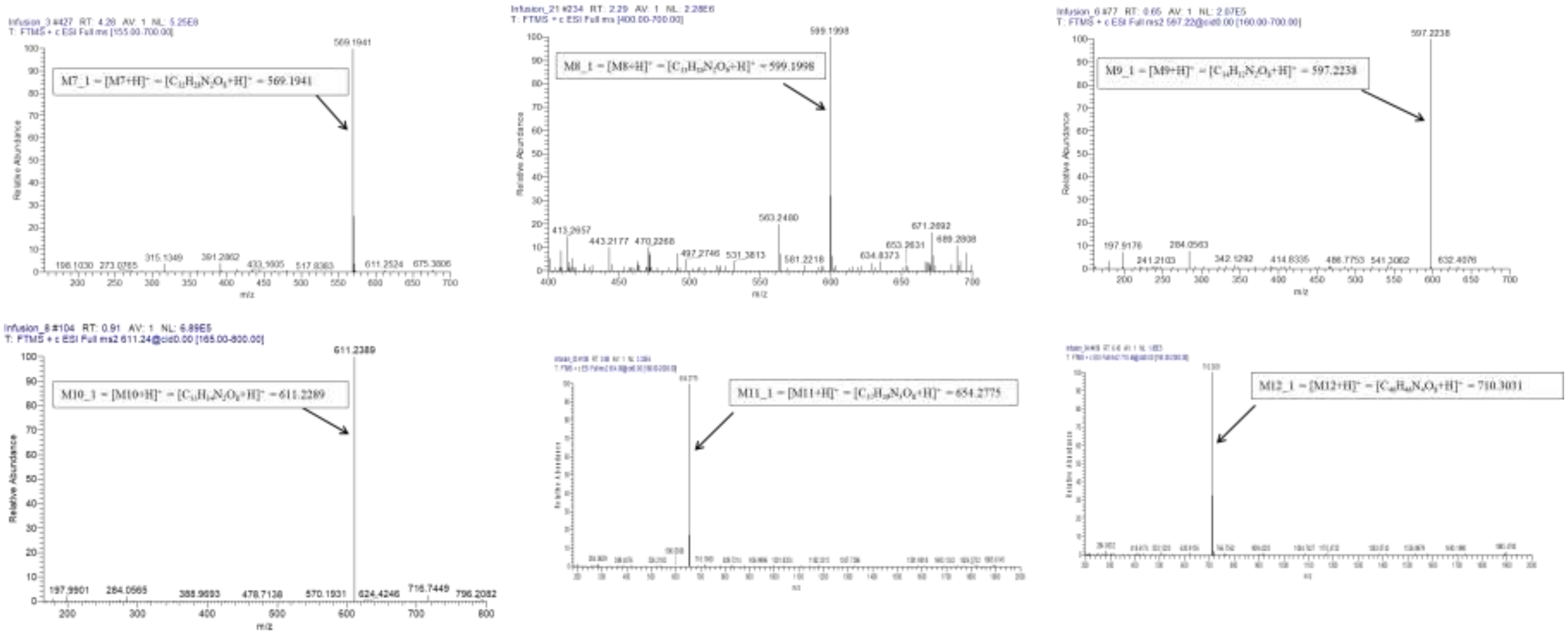

Figure S2. ESI-MS spectra of Naringenin derivatives recorded in positive mode

Table S1. Absolute error calculations between m/z values of ESI-MS spectrometric measurements (Experimental) and theoretical calculations (simulated values)

Species Formula	Experimental values	Simulated values*	Absolute errors
M1_1=$[M1]^+$=$C_{56}H_{68}N_2O_{26}$	1185.4489	1185.4143	0.0346
M1_2=$[M1-C_{27}H_{33}O_{13}]^+$=$[C_{29}H_{35}N_2O_{13}+4H]^+$=$[C_{29}H_{39}N_2O_{13}]^+$	623.2611	623.2447	0.0164
M2_1=$[M2]^+$=$[C_{57}H_{70}N_2O_{27}]^+$	1215.4283	1215.4239	0.0044
M3_1=$[M3]^+$=$[C_{58}H_{72}N_2O_{26}]^+$	1213.4441	1213.4446	0.0005
M4_1=$[M4]^+$=$[C_{59}H_{74}N_2O_{26}]^+$	1227.4692	1227.4603	0.0089
M5_1=$[M5]^+$=$[C_{61}H_{79}N_3O_{26}]^+$	1270.5035	1270.4980	0.0055
M6_1=$[M6]^+$=$[C_{64}H_{86}N_4O_{26}]^+$	1327.5599	1327.5603	0.0004
M7_1=$[M7+H]^+$=$[C_{32}H_{28}N_2O_8+H]^+$	569.1941	569.1918	0.0023
M8_1=$[M8+H]^+$=$[C_{33}H_{30}N_2O_9+H]^+$	599.1998	599.2024	0.0026
M9_1=$[M9+H]^+$=$[C_{34}H_{32}N_2O_8+H]^+$	597.2238	597.2231	0.0007
M10_1=$[M10+H]^+$=$[C_{35}H_{34}N_2O_8+H]^+$	611.2289	611.2388	0.0099
M11_1=$[M11+H]^+$=$[C_{37}H_{39}N_3O_8+H]^+$	654.2775	654.281	0.0035
M12_1=$[M12]^+$=$[C_{40}H_{46}N_4O_8]^+$	710.3031	710.331	0.0279

* All simulated values have been calculated using the Xcalibur software

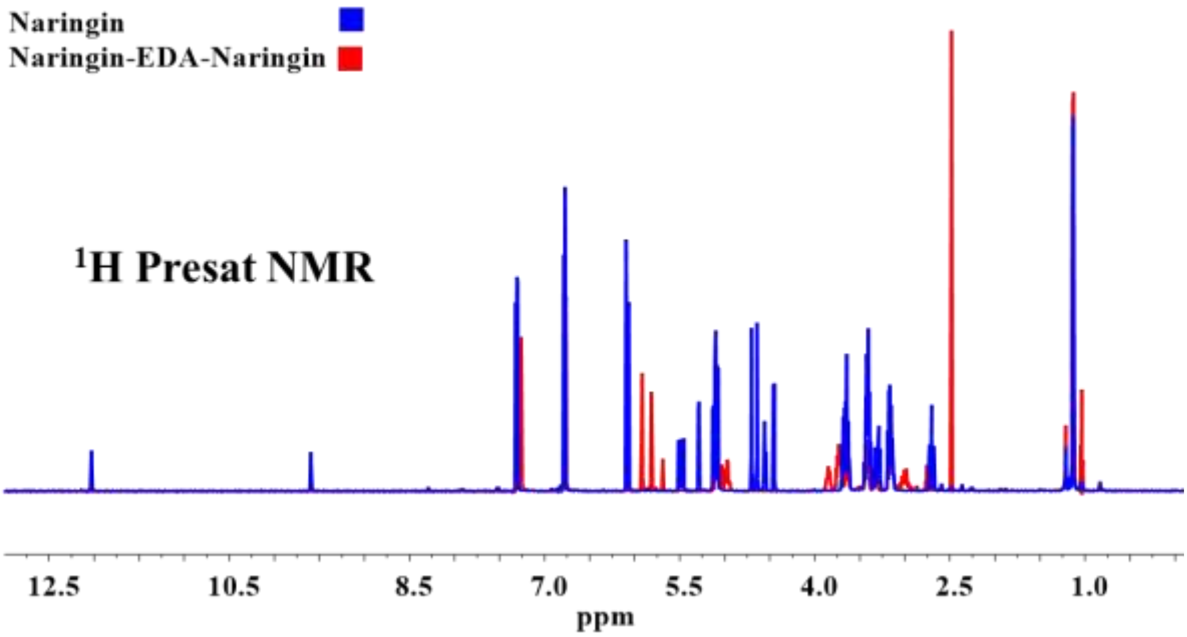

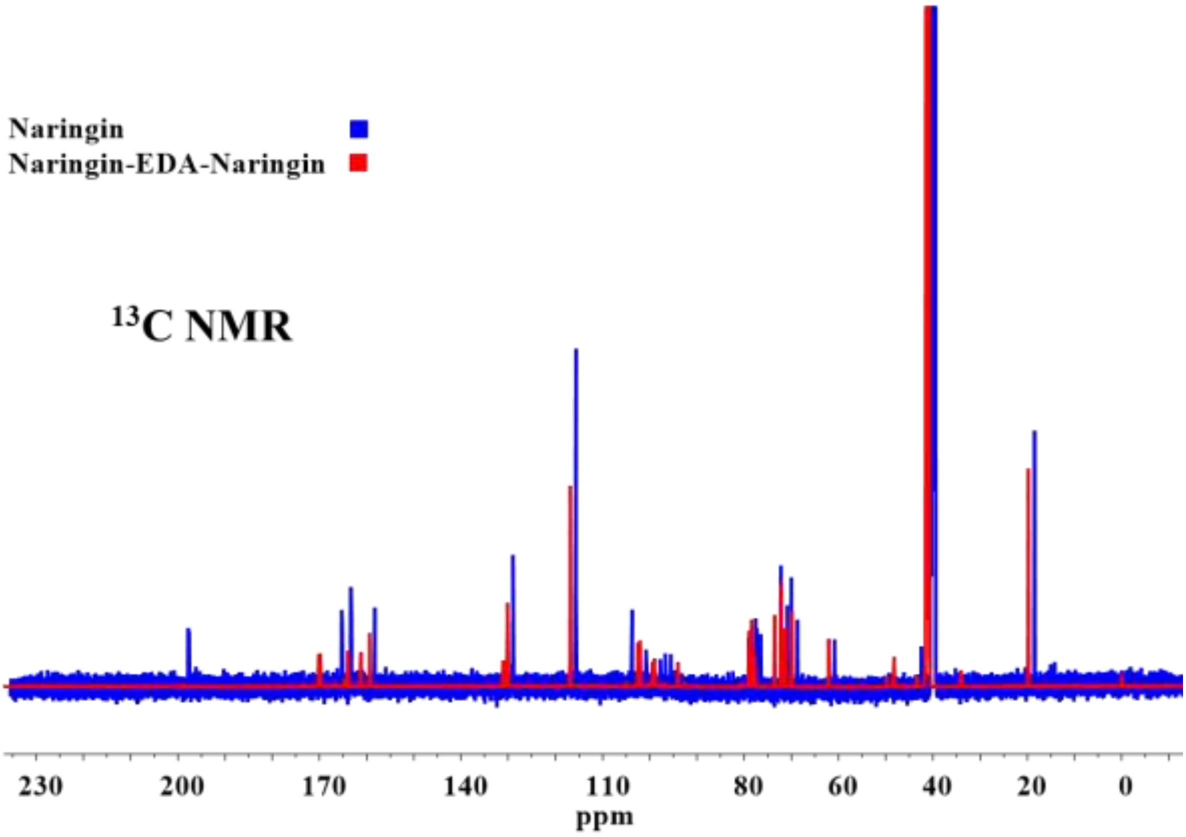

Figure S3. Comparative ^{1}H Presat and ^{13}C NMR spectra of modified Naringin-EDA-Naringin with free Naringin in DMSO-d^{6}

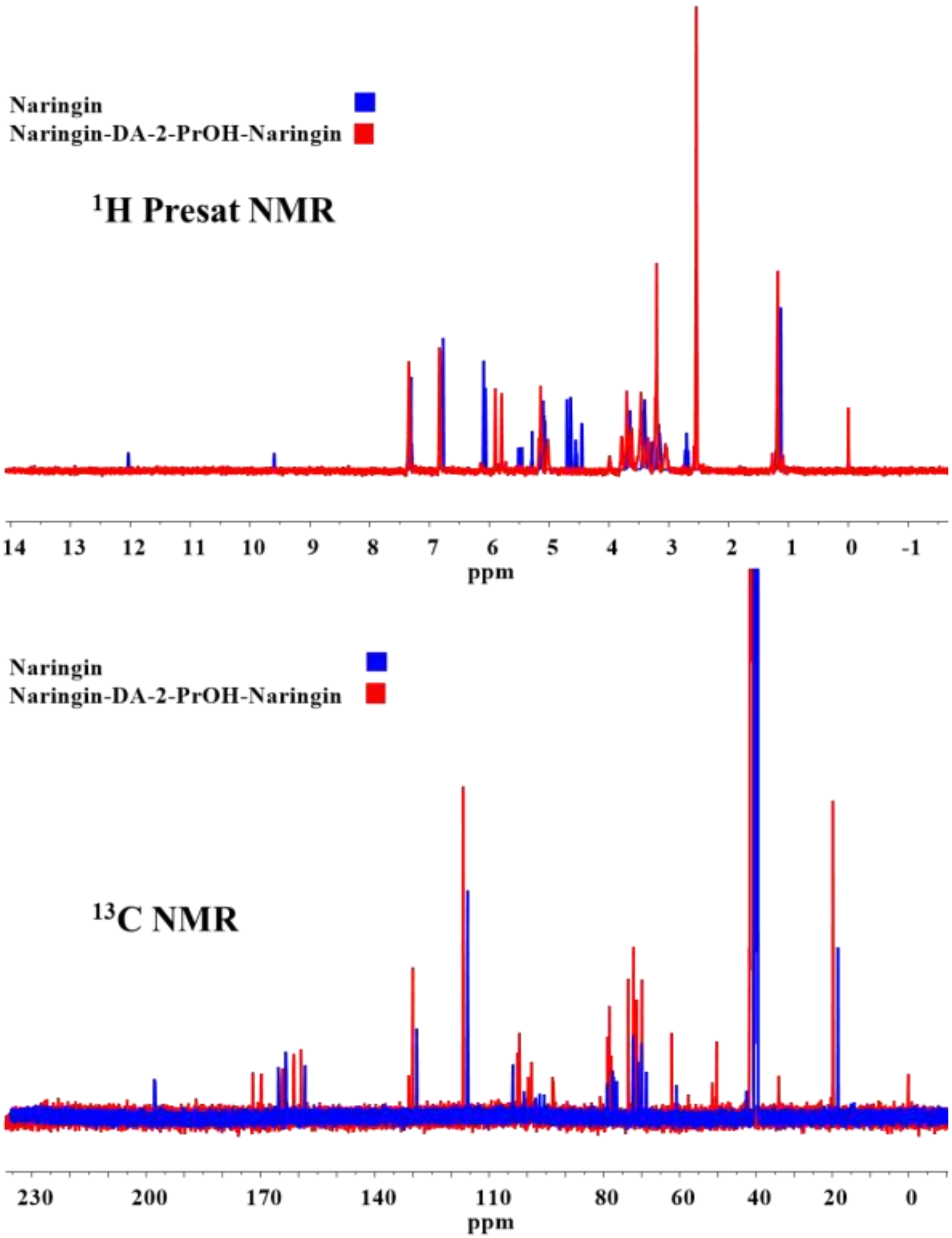

Figure S4. Comparative ¹H Presat and ¹³C NMR spectra of modified Naringin-DA-2-PrOH-Naringin with free Naringin in DMSO-d⁶

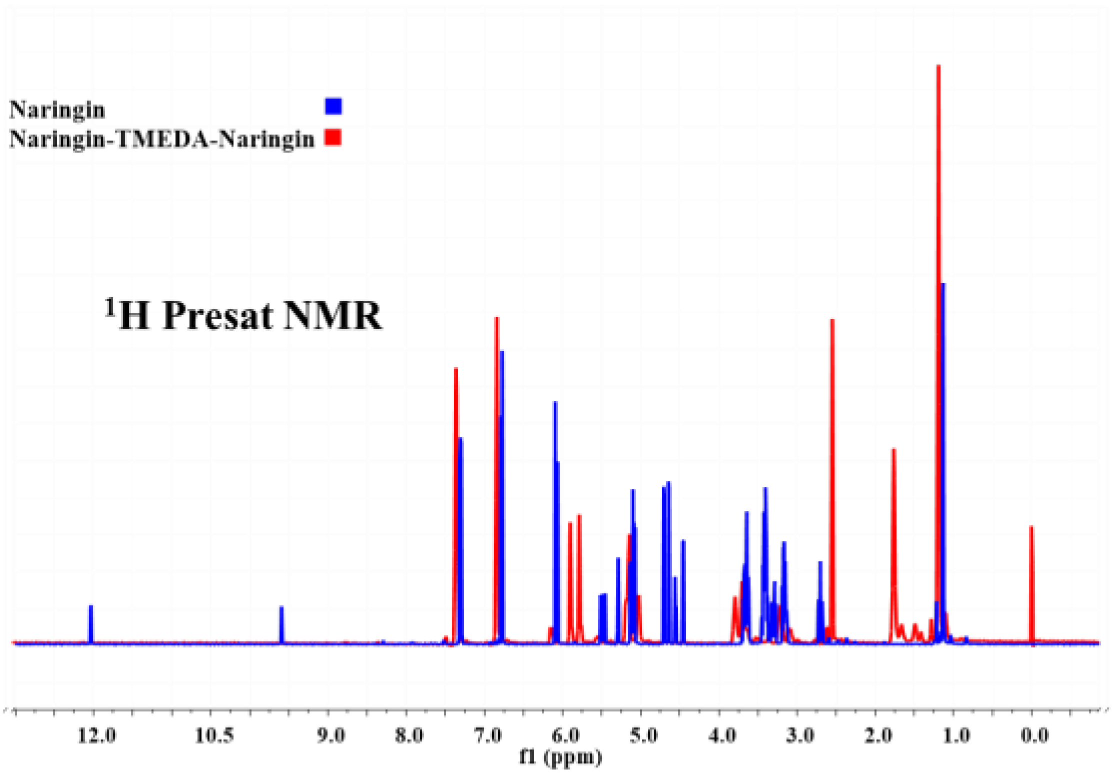

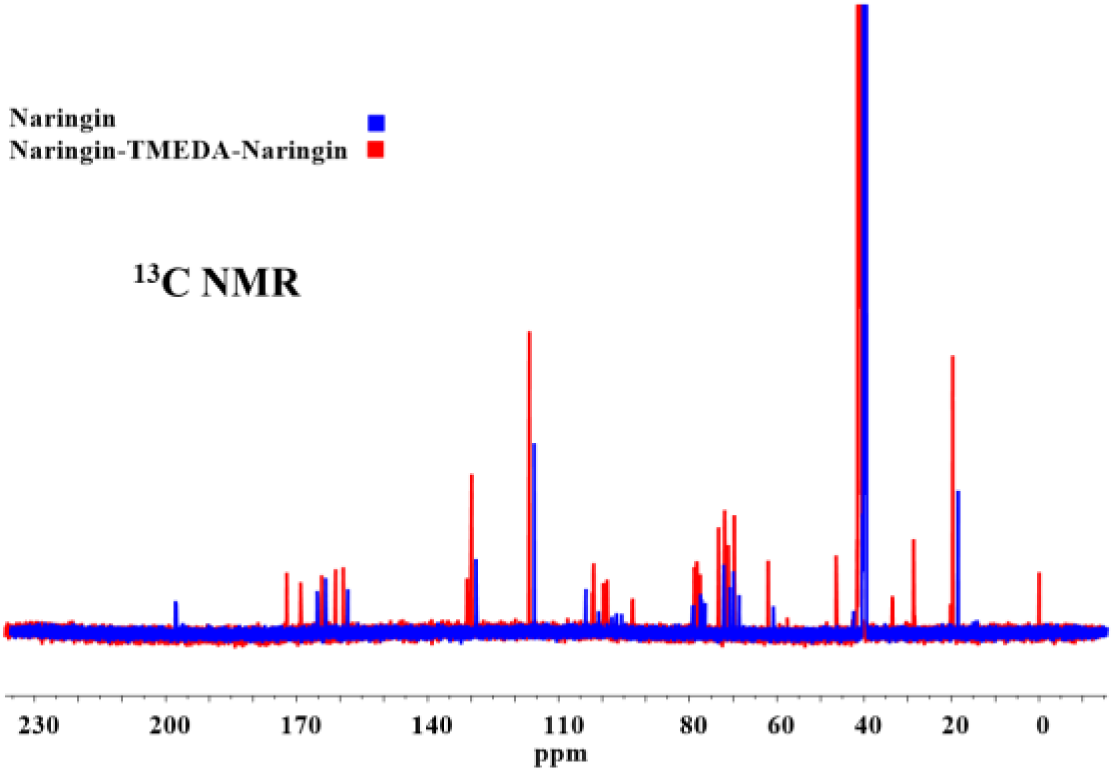

Figure S5. Comparative ^{1}H Presat and ^{13}C NMR spectra of modified Naringin-TMEDA-Naringin with free Naringin in DMSO-d^6

^{13}C NMR

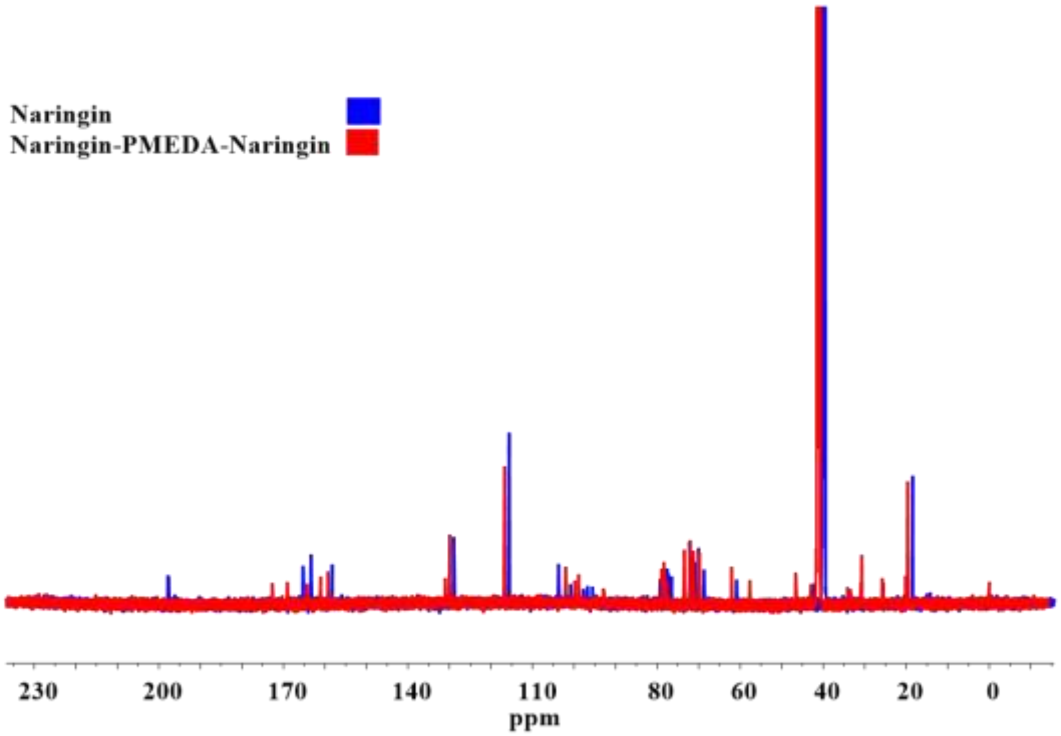

Figure S6. Comparative ^{13}C NMR spectra of modified Naringin-PMEDA-Naringin with free Naringin in DMSO-d^6

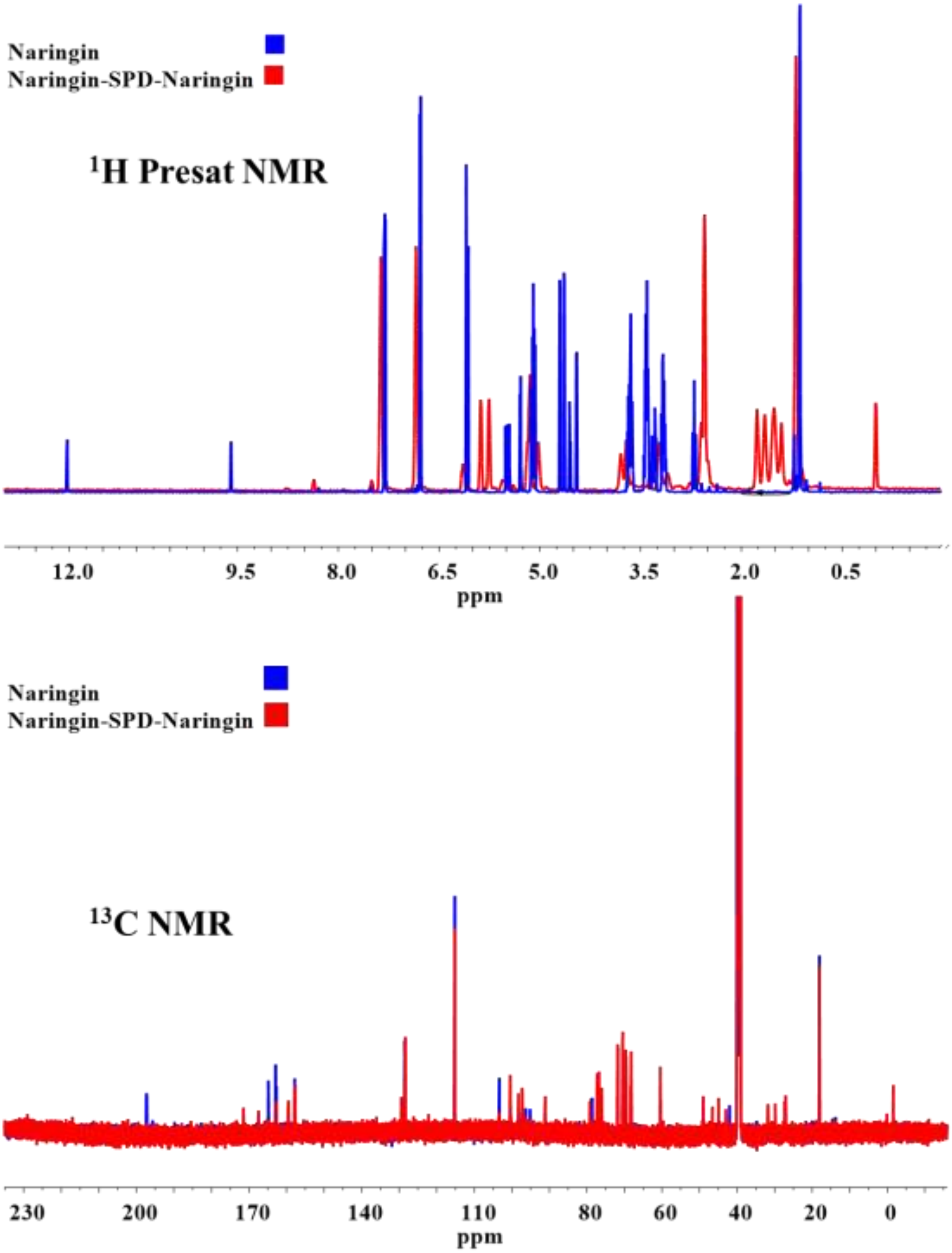

Figure S7. Comparative ^{1}H Presat and ^{13}C NMR spectra of modified Naringin-SPD-Naringin with free Naringin in DMSO-d^{6}

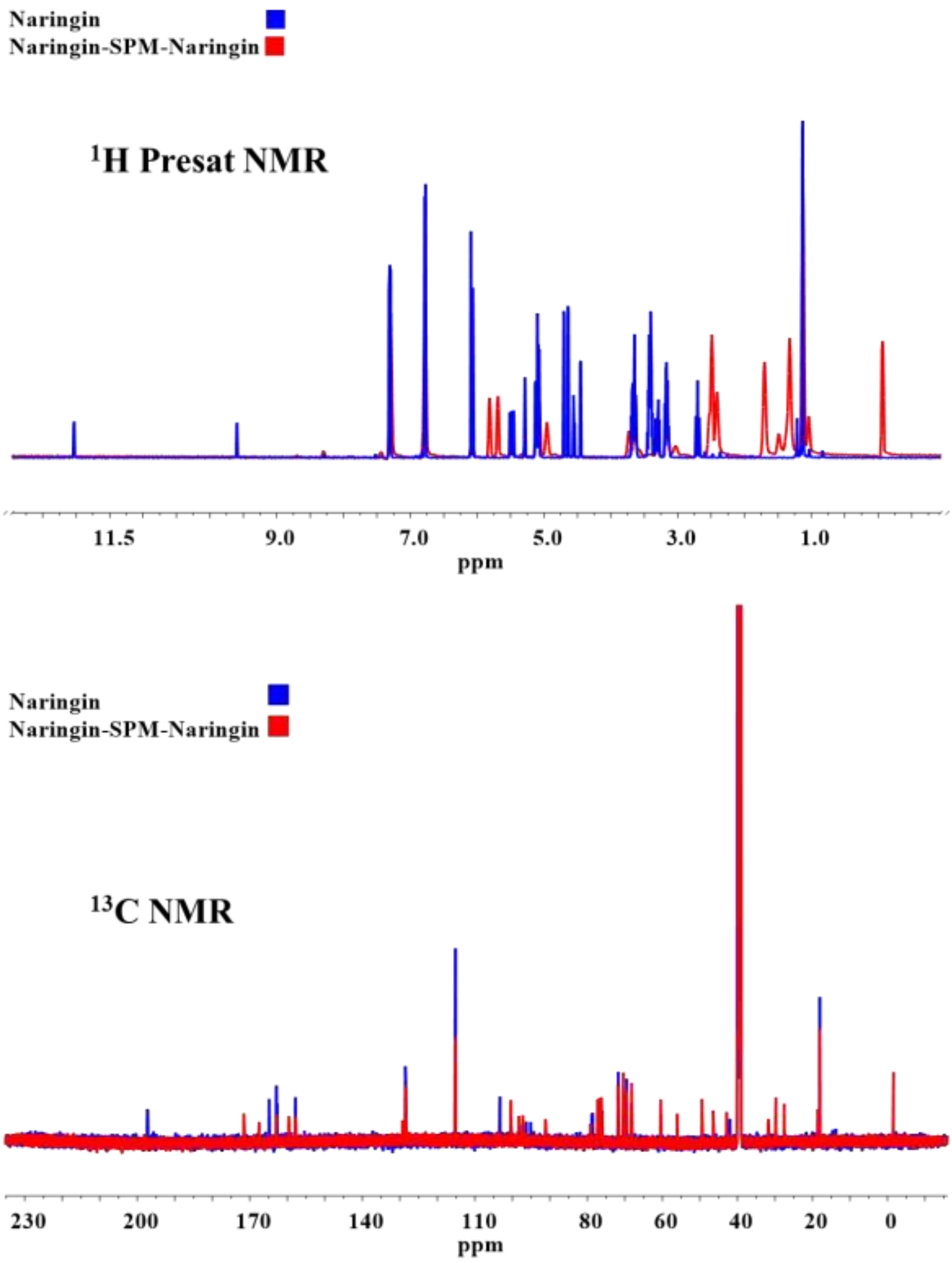

Figure S8. NMR Comparative ^{1}H Presat and ^{13}C NMR spectra of modified Naringin-SPM-Naringin with free Naringin in DMSO-d[6]

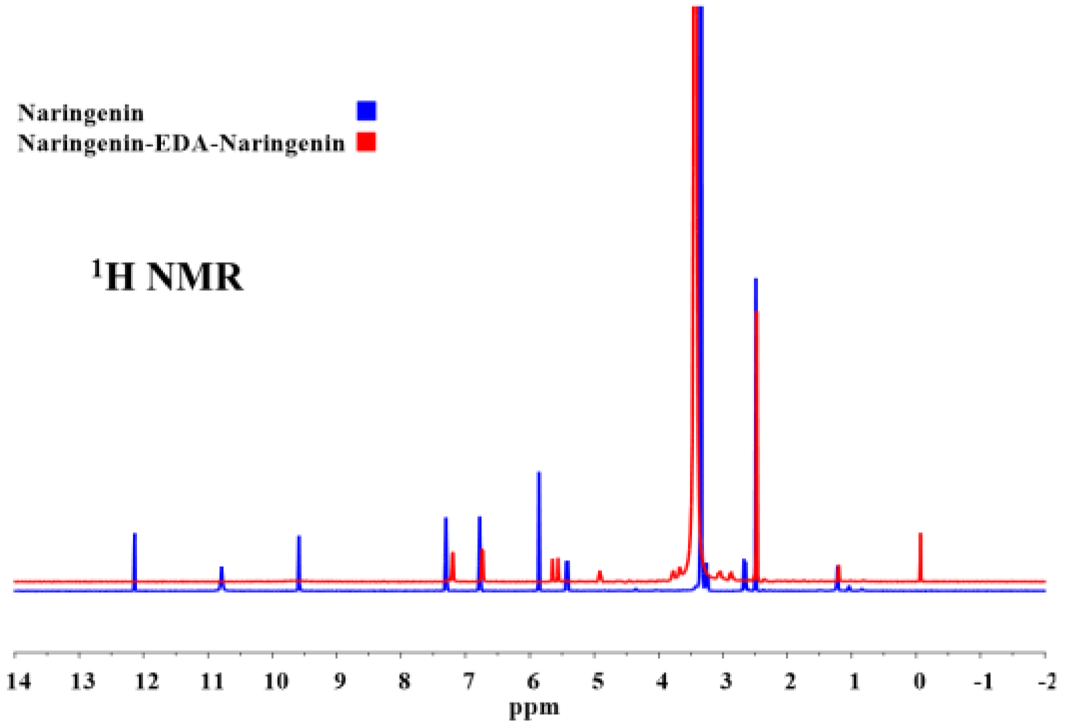

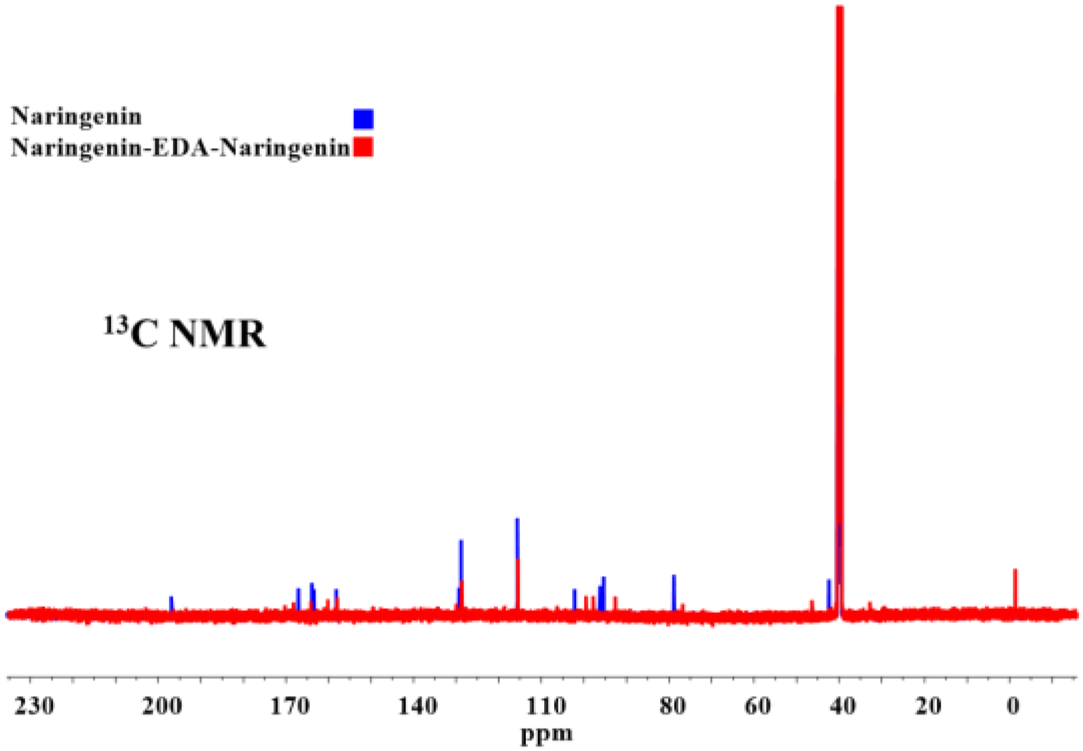

Figure S9. Comparative ${}^{1}H$ Presat and ${}^{13}C$ NMR spectra of modified Naringenin-EDA-Naringenin with free Naringenin in DMSO-d^6

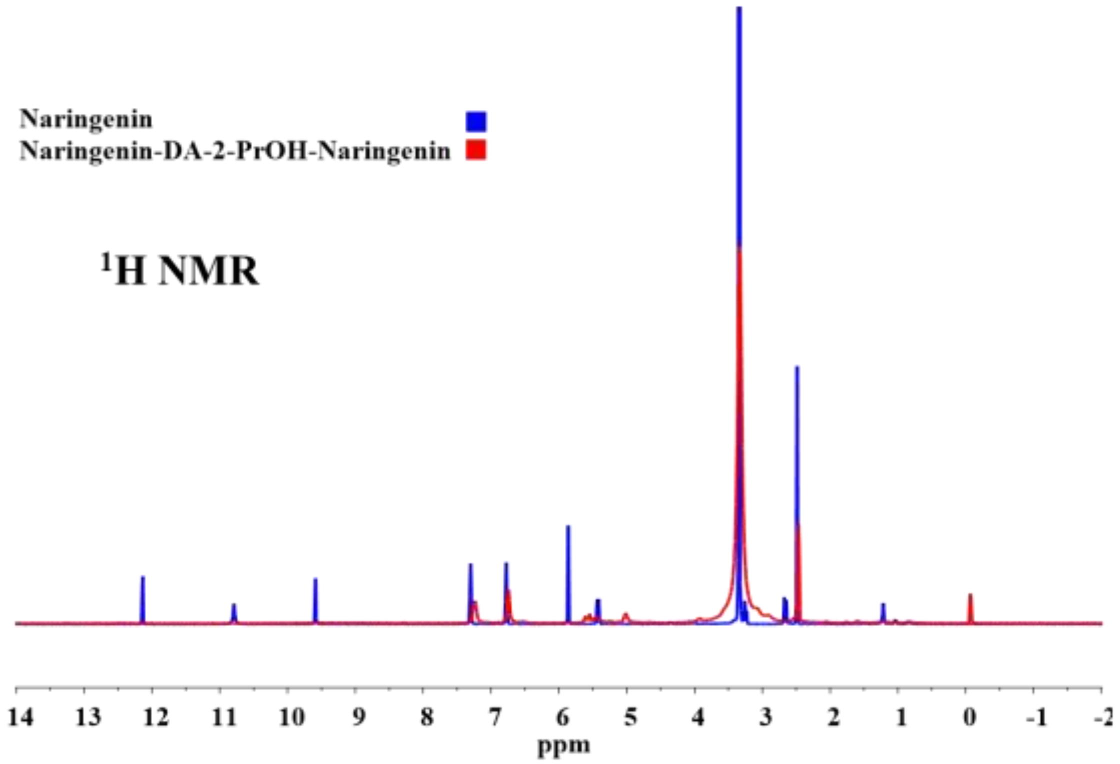

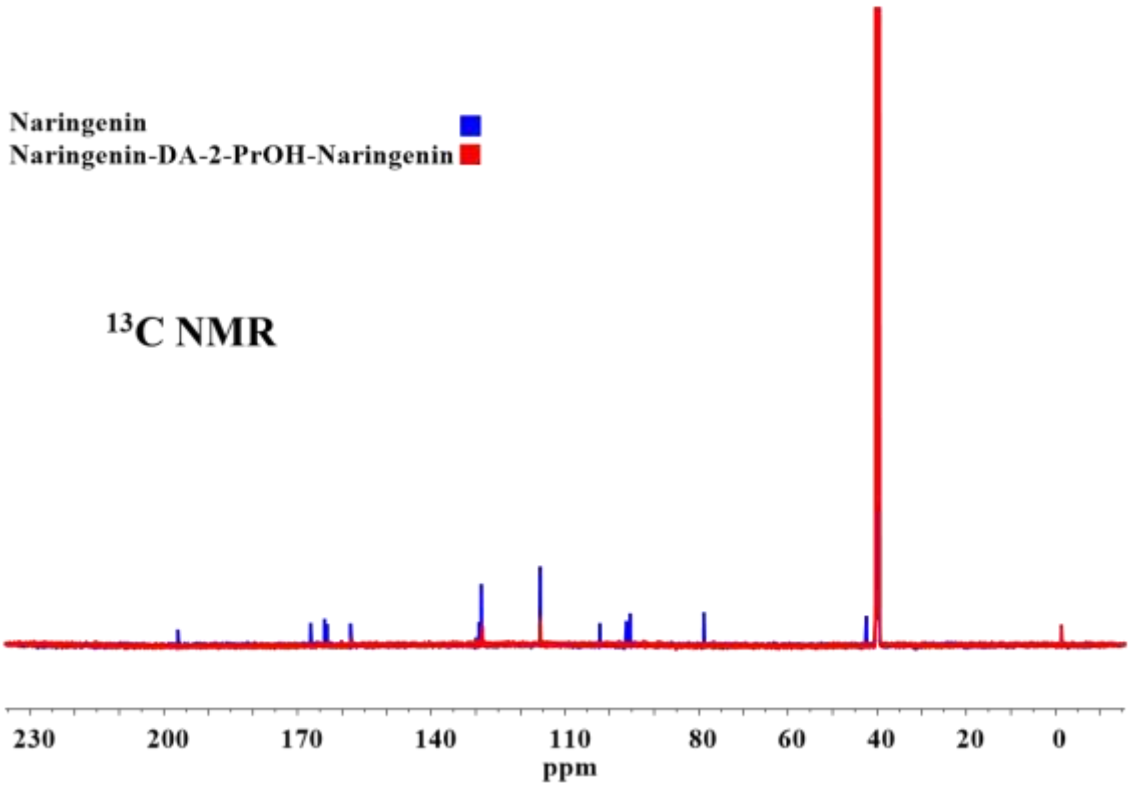

Figure S10. Comparative ^{1}H Presat and ^{13}C NMR spectra of modified Naringenin-DA-2-PrOH-Naringenin with free Naringenin in DMSO-d^6

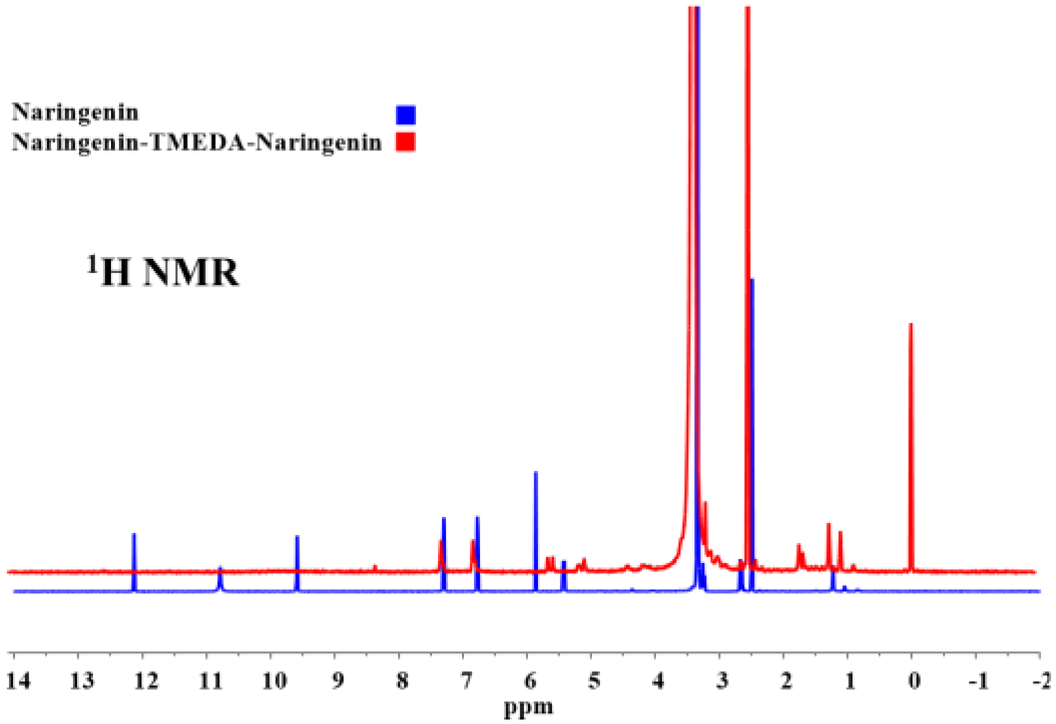

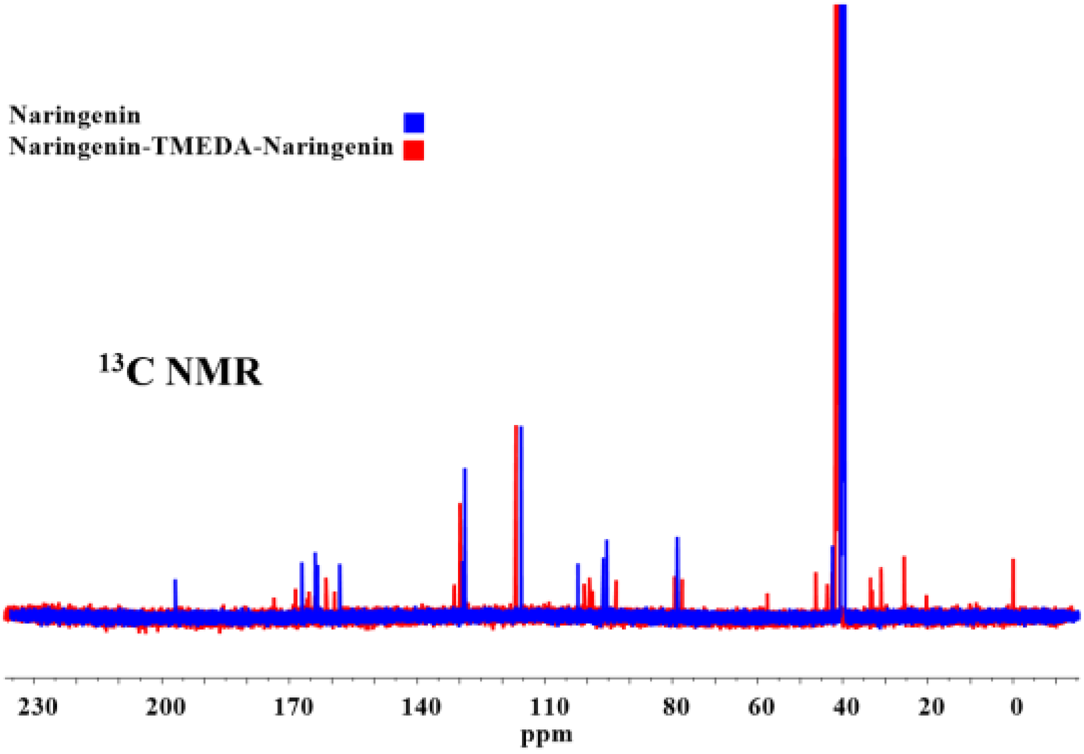

Figure S11. Comparative ^{1}H Presat and ^{13}C NMR spectra of modified Naringenin-TMEDA-Naringenin with free Naringenin in DMSO-d^{6}

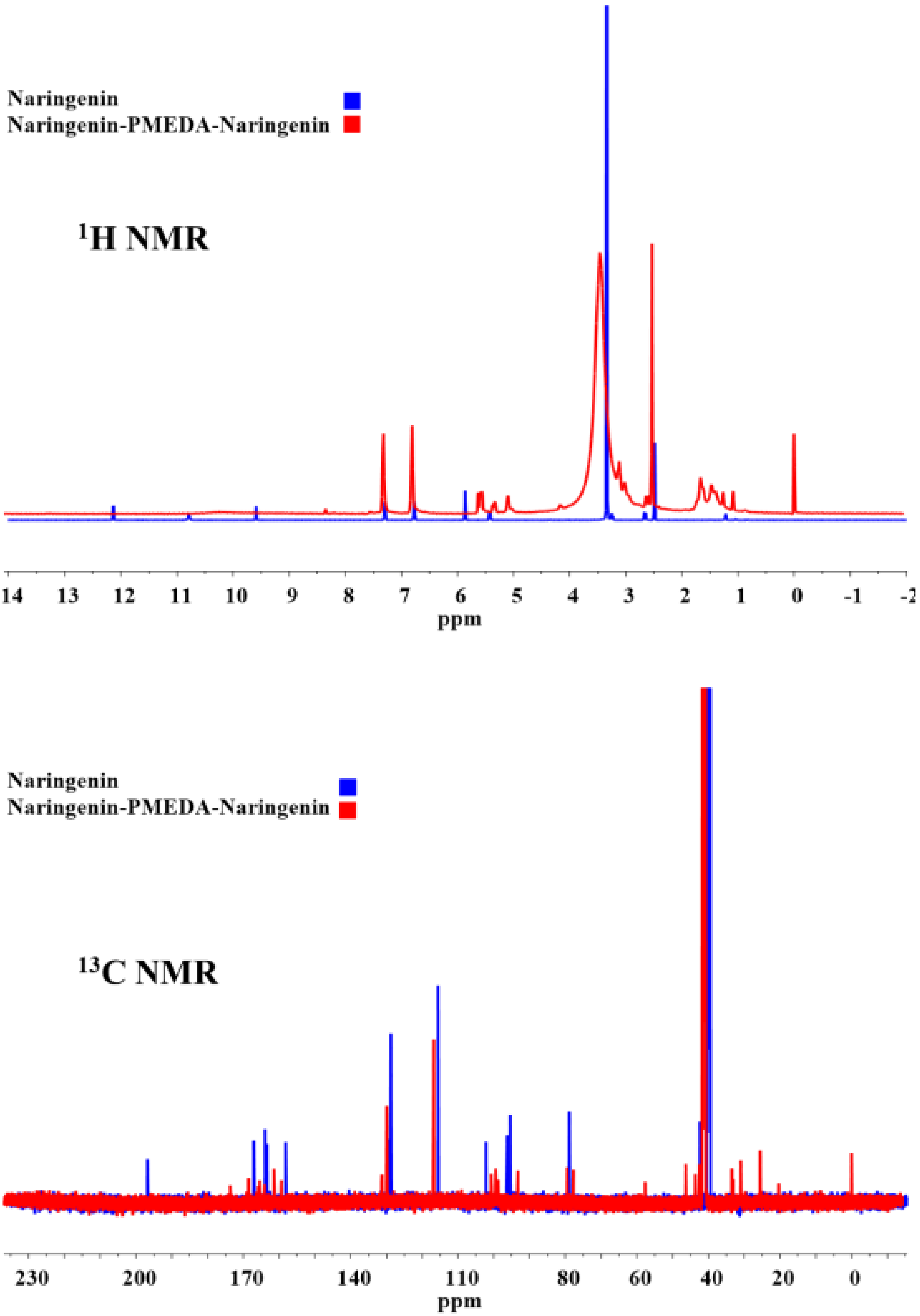

Figure S12. Comparative ^{1}H Presat and ^{13}C NMR spectra of modified Naringenin-PMEDA-Naringenin with free Naringenin in DMSO-d^{6}

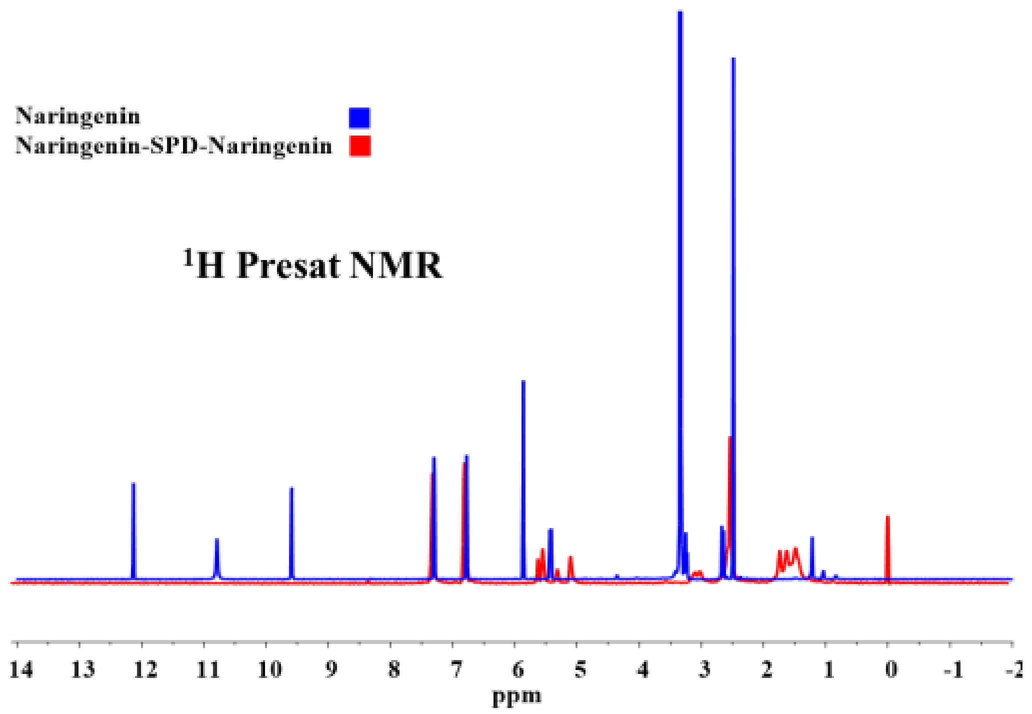

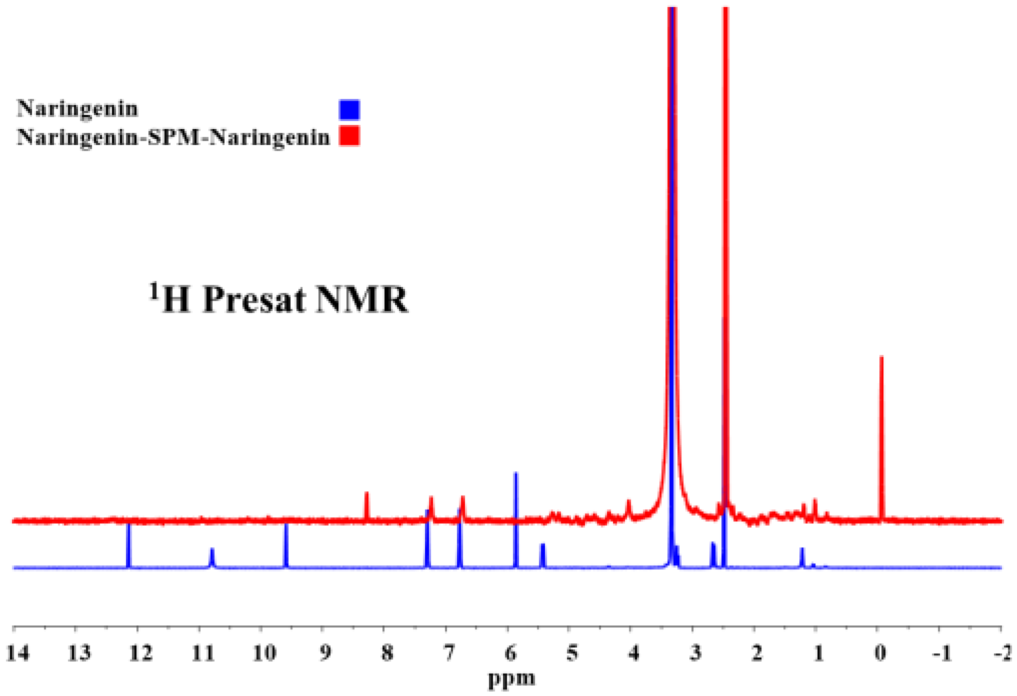

Figure S13. Comparative ^{1}H Presat spectra of modified Naringenin-SPD-Naringenin and Naringenin-SPM-Naringenin with free Naringenin in DMSO-d^{6}

Note: Due to low solubility of Naringenin-SPM-Naringenin the carbon spectrum was not recorded.
